# COSMOS next generation – A public knowledge base leveraging chemical and biological data to support the regulatory assessment of chemicals

**DOI:** 10.1016/j.comtox.2021.100175

**Published:** 2021-08

**Authors:** C. Yang, M.T.D. Cronin, K.B. Arvidson, B. Bienfait, S.J. Enoch, B. Heldreth, B. Hobocienski, K. Muldoon-Jacobs, Y. Lan, J.C. Madden, T. Magdziarz, J. Marusczyk, A. Mostrag, M. Nelms, D. Neagu, K. Przybylak, J.F. Rathman, J. Park, A-N Richarz, A.M. Richard, J.V. Ribeiro, O. Sacher, C. Schwab, V. Vitcheva, P. Volarath, A.P. Worth

**Affiliations:** a MN-AM, Columbus, OH, USA; b MN-AM Nürnberg, Germany; c School of Pharmacy and Biomolecular Sciences, Liverpool John Moores University, UK; d US FDA CFSAN College Park, MD, USA; e Cosmetic Ingredient Review, Washington, DC, USA; f University of Bradford, UK; g The Ohio State University, Columbus OH, USA; h US EPA RTP, NC, USA; i European Commission, Joint Research Centre (JRC), Ispra, Italy

**Keywords:** AOP, Adverse Outcome Pathway, CERES, Chemical Evaluation and Risk Estimation System, CFSAN, Center for Food Safety and Applied Nutrition, CosIng, Cosmetic Ingredient Database, COSMOS DB, COSMOS Database, CMS-ID, COSMOS Identification Number, COSMOS MINIS, Minimum Inclusion Criteria of Studies in COSMOS DB, COSMOS NG, COSMOS Next Generation, CRADA, Cooperative Research and Development Agreement, DART, Developmental & Reproductive Toxicity, DB, Database, DST, Dempster Shafer Theory, ECHA, European Chemicals Agency, EFSA, European Food Safety Authority, HESS, Hazard Evaluation Support System, HNEL, Highest No Effect Level, HTS, High throughput screening, LEL, Lowest Effect Level, LogP, Logarithm of the octanol:water partition coefficient, ILSI, International Life Sciences Institute, LOAEL, Lowest Observed Adverse Effect Level, IUCLID, International Uniform Chemical Information Database, NAM, New Approach Methodology, NGRA, Next Generation Risk-Assessment, NITE, National Institute of Technology and Evaluation (Japan), NOAEL, No Observed Adverse Effect Level, NTP, National Toxicology Program, OECD, Organisation for Economic Co-operation and Development, OpenFoodTox, EFSA’s OpenFoodTox database, PAFA, Priority-based Assessment of Food Additive database, PK/TK, Pharmacokinetics/Toxicokinetics, QA, Quality Assurance, QC, Quality Control, REACH, Registration, Evaluation, Authorisation and Restriction of Chemicals, SCC, Science Committee on Cosmetics (EU), SCCS, Scientific Committee on Consumer Safety (EU), SCCP, Scientific Committee on Consumer Products (EU), SCCNFP, Scientific Committee of Cosmetic Products and Non-food Products intended for Consumers (EU), ToxRefDB, Toxicity Reference Database, TTC, Threshold of Toxicological Concern, US EPA, United States Environmental Protection Agency, US FDA, United States Food and Drug Administration, Toxicity, Database, Public database, Knowledge hub, Study reliability, Analogue selection, Guided workflow

## Abstract

The COSMOS Database (DB) was originally established to provide reliable data for cosmetics-related chemicals within the COSMOS Project funded as part of the SEURAT-1 Research Initiative. The database has subsequently been maintained and developed further into COSMOS Next Generation (NG), a combination of database and *in silico* tools, essential components of a knowledge base. COSMOS DB provided a cosmetics inventory as well as other regulatory inventories, accompanied by assessment results and *in vitro* and *in vivo* toxicity data. In addition to data content curation, much effort was dedicated to data governance – data authorisation, characterisation of quality, documentation of meta information, and control of data use. Through this effort, COSMOS DB was able to merge and fuse data of various types from different sources. Building on the previous effort, the COSMOS Minimum Inclusion (MINIS) criteria for a toxicity database were further expanded to quantify the reliability of studies. COSMOS NG features multiple fingerprints for analysing structure similarity, and new tools to calculate molecular properties and screen chemicals with endpoint-related public profilers, such as DNA and protein binders, liver alerts and genotoxic alerts. The publicly available COSMOS NG enables users to compile information and execute analyses such as category formation and read-across. This paper provides a step-by-step guided workflow for a simple read-across case, starting from a target structure and culminating in an estimation of a NOAEL confidence interval. Given its strong technical foundation, inclusion of quality-reviewed data, and provision of tools designed to facilitate communication between users, COSMOS NG is a first step towards building a toxicological knowledge hub leveraging many public data systems for chemical safety evaluation. We continue to monitor the feedback from the user community at support@mn-am.com.

## Highlights

• The historical development of the public COSMOS database is provided.
• COSMOS NG is a knowledge hub to share toxicity data and *in silico* tools.
• COSMOS NG has broad chemical coverage, with a focus on cosmetics.
• Chemical and toxicological data are quality assured through inclusion criteria.
• In silico TTC, profiling and read-across workflows are illustrated.

## Introduction

### COSMOS DB as a platform for leveraging public resources

The European Union’s Cosmetics Regulation (EC) N° 1223/2009 entered into force in January 2010, maintaining the provisions of the Seventh Amendment of the European Union’s Cosmetics Directive 76/768/EEC, foreseeing the ultimate replacement of animal testing of cosmetic products for all endpoints, including repeated dose/reproductive toxicity and toxicokinetics; the full EU ban on animal testing for cosmetics entered into force in March 2013 [1]. To this end, the SEURAT-1 Research Initiative [2] was funded from 2011 to 2016 by the European Commission (7th European RTD Framework Programme; FP7) and the cosmetics industry (Cosmetics Europe) [3], [4]. The COSMOS Project was one of seven projects in the cluster, which was initiated to fill the gap in knowledge and technology based on *in silico* toxicology [5]. The management and sharing of chemical, biological and toxicity data are critical capabilities underpinning this effort. The COSMOS DB [6] was designed to service the work packages pertaining to non-testing and/or computational methods, including Threshold of Toxicological Concern (TTC), read-across, quantitative structure–activity relationship (QSAR) models, biokinetics, and innovative chemistry methods. To support these activities, the compilation and curation of vastly different types of data content enriched with cosmetics-related chemicals to be made publicly available in a database format were necessary. In addition, this project had a unique opportunity to include the US Food and Drug Administration (FDA) Center for Food Safety and Applied Nutrition (CFSAN) as a partner and US Environmental Protection Agency (EPA) National Center for Computational Toxicology (now part of the Center for Computational Toxicology & Exposure) as a member of the scientific advisory board. The public databases released from the two agencies were a tremendous jump start for the project.

The Chemical Evaluation and Risk Estimation (CERES) [7] project at FDA CFSAN has the CERES database as a core component of its risk assessment system. CERES contains CFSAN-related regulatory information (historical as well as current information). CERES also houses other toxicity databases, including the legacy Priority-based Assessment of Food Additive database (PAFA) [8], containing both chemical safety records and toxicity data enriched with food ingredients and additives, as well as chemicals related to food packaging that are more closely related to cosmetics. The CERES team has been among the essential contributors to and users of the ToxML standard [10] and has continued their well-structured database activities for nearly two decades. Much of CFSAN’s publicly available data has been collected using the ToxML data standard and shared with various commercial collaborators under CRADAs (Cooperative Research and Development Agreement) [9], Research Collaboration Agreements, and Data Transfer Agreements. The CERES database has also functioned as the core resource-sharing platform with other non-commercial initiatives such as ToxCast [11], Tox21 [12], and EU projects. To promote collaboration between COSMOS and FDA CFSAN, the legacy PAFA database along with other publicly available data from the CERES [7] database was donated to COSMOS in 2013 and updates have been regularly shared since. The two databases, COSMOS DB v1 and CERES, were based on similar technology approaches, including the architecture and schema. In addition to the public content of the CERES database, the software user interface, developed for applying the design suggestions from reviewers at FDA CFSAN, was also part of the donation to COSMOS DB v1.

It was essential that the chemistry content in COSMOS DB should include inventories from cosmetics ingredients and related substances used in cosmetics products, as well as other relevant chemicals such as fragrances, food additives, and colourants. For biological data, it was imperative to abstract the content with a logical data model designed to accommodate vastly different information types, e.g., oral repeated-dose toxicity, skin permeability data, pharmacokinetic (PK) and toxicokinetic (TK) studies, and metabolism information. Whilst accommodating the different types of domain knowledge was essential, it was also important that the data conform to existing toxicity database standards such as ToxML or the harmonised templates (OHT) [13] from the Organisation for Economic Co-operation and Development (OECD) [14]. Such standardisation would facilitate data exchanges with other external databases and projects.

One of the most critical considerations in building COSMOS DB was the lack of available data in the public domain for cosmetics-related chemicals. There have been various public database projects that have been constructed to consolidate toxicity data for regulatory use or modelling studies, such as Ambit [15], OpenFoodTox [16], [17], ToxRefDB [18], HESS [19], or RepDose [20] to name a few. Although most of these public projects, wholly or in part, were open-source with free access, the chemical space did not provide adequate coverage of cosmetics- or food ingredients-related chemicals. Therefore, the requirements of COSMOS DB were to: 1) compile cosmetics-related chemicals listed in public resources; 2) gather regulatory information (such as daily intake estimates or regulation history) on as many of these chemicals as possible; 3) store detailed biological data at the dose/concentration level; 4) provide capabilities for searching/browsing and reporting of the data for regulatory use cases; 5) adopt open-source technology and a public database model so that the implementation would not depend on proprietary back-end technology. In summary, the construction of the new COSMOS DB was deemed necessary since, except in the case of the EU CosIng database [21] linked with Scientific Committee of Consumer Safety (SCCS) opinions [22], there was no other public resource to query whether a chemical had been used for cosmetics products or whether any biological data were available. For this reason, the COSMOS project created a consolidated forum for hosting data on cosmetics-related chemicals, accompanied by the status of the affiliated experimental data and regulatory information. Since the official release of the COSMOS DB v2 in 2015, over 3000 users are registered to access the database, with 75% of these users from the commercial industrial sector.

The COSMOS DB v2 has been further developed and continuously maintained by MN-AM (Molecular Networks – Nürnberg, Germany, and Altamira LLC, Columbus, OH, USA) to host on-line access, with new data and features added on a regular basis. Liverpool John Moores University (LJMU), the project coordinator of the COSMOS project, has also continuously participated in this effort to the present, beyond the official completion of the COSMOS project in December of 2015. During the following five years, the public landscape of data resources and *in silico* methodologies changed significantly; one of many examples is the CompTox Chemicals Dashboard provided by the US EPA [23]. Reflecting these developments, COSMOS DB has now been expanded to COSMOS Next Generation (COSMOS NG) [24] to offer high quality data from diverse experimental studies and interpretable *in silico* methodologies. This new system allows users to calculate molecular / physicochemical properties and to profile compounds with publicly available chemical categories and structural alerts. Building on existing functionality for similarity and substructure searching to find analogues, the system’s capabilities are further strengthened since the chemoinformatics platform was designed to support the tasks necessary for a read-across workflow. This paper describes a step-by-step guided workflow for read-across using the data and tools provided by COSMOS NG. In addition, the public component of the ChemTunes web services [25] integrated within COSMOS NG will also include the previously available Physiologically based Kinetic (PBK) models originally developed for the COSMOS Project [5]. Beyond the utility of these new features, the ultimate value of the new COSMOS NG could be as a knowledge hub, through which the direct communications between users from multiple institutions and with different roles is enabled.

**Fig. 1:**
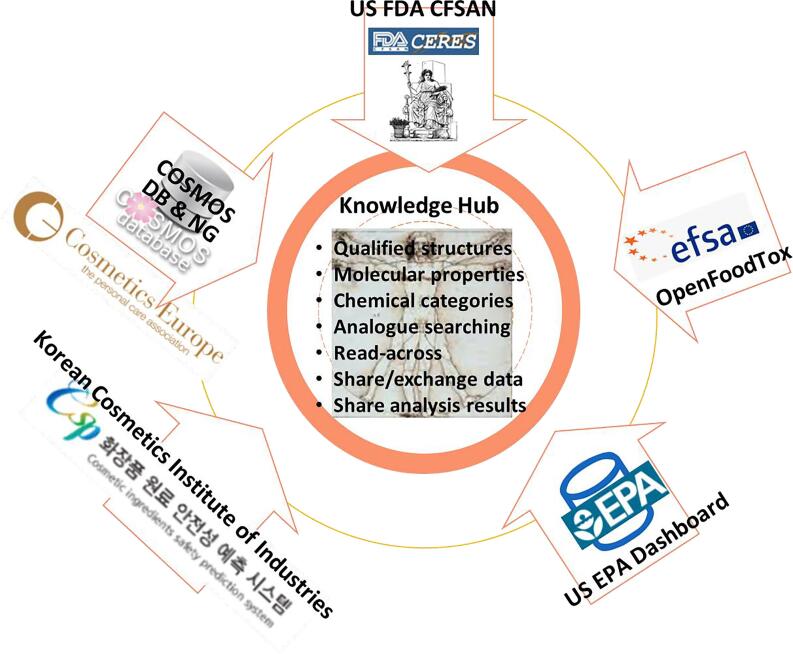
Conceptual Framework of a Knowledge Hub Leveraging Public Resources.

The fully developed system would leverage public resources to offer a unique knowledge hub based on COSMOS NG, extending capabilities beyond COSMOS DB. Technically, a knowledge hub requires a registry of various tools and systems with which the data and knowledge will be exchanged, adaptors for each data sources, methods to process workflows, and means to communicate results with the desired receivers. Scientifically, it should provide quality structures and means to calculate molecular / physicochemical properties, map structures to chemical categories and alerts, and predict important endpoints of toxicity and kinetics. The system will eventually become a true knowledge hub, a centre for many new paradigms in holistic *in silico* chemical safety assessment needed to support next generation risk assessment (NGRA) [26], [27]. Furthermore, the system would allow for the sharing of the assessment results among users pending the access status. We envision an *in silico* assessment with the results being shared between industry and regulatory agencies through the knowledge hub. Fig. 1 summarises the conceptual framework.

This paper describes the evolution of COSMOS DB (from FDA’s original donated version of the CERES software and corresponding publicly available data to COSMOS DB v1 and v2) and its transformation to COSMOS NG to firmly establish a knowledge base and foundation for an eventual knowledge hub. To this end, the paper is organised into the following sections: 1) the current key features of the COSMOS DB (Compound Information, Safety Evaluation, Toxicity data); 2) database process and content expansion of the current *in vivo* and *in vitro* data types along with data quality measures - the database architecture is designed such that new data types can include data from new approaches such as toxicogenomics and transcriptomics; 3) COSMOS NG workflow tools to further analyse similarity measures, calculate molecular / physiochemical properties, profile chemicals for categories and alerts, and execute the current TTC decision tree; 4) the potential role of COSMOS NG as a knowledge hub, with a demonstration of a sharable guided workflow to assist users in applying data and knowledge from COSMOS NG in a read-across application.

## Ceres and COSMOS databases

### Overview of database technology

#### Architecture and data model of CERES and COSMOS databases

The US FDA CFSAN CERES Database, whose public content was donated to COSMOS v1 in 2012, contained over 82,000 substances and ~ 55,000 unique structures. Each substance was registered with an identifier of “CRS”. The donated CERES database (COSMOS v1) acted as a central resource providing reviewer access to data from several regulatory programs within CFSAN, as well as the PAFA legacy database. COSMOS v1 provided 69,911 compounds with “CMS” as identifiers corresponding to 44,576 total structures (with connection tables from public sources). CRS and CMS identifiers were all matched for the COSMOS DB versions. Each substance goes through curation for establishing a CRS-ID, CAS RN, and preferred name. An instance of COSMOS DB v1 was hosted from University of Bradford (UK) for public access whereas internal collaboration version was hosted at the Amazon Webservice (AWS) during the COSMOS Project. The content of the v1 database is available from the COSMOS Project website [5] or as downloadable datasets within COSMOS NG. At the end of the project duration, COSMOS DB v2 was migrated to MN-AM for hosting, maintenance, and further development.

CERES and COSMOS databases shared common technical and scientific foundations. COSMOS DB v1 was a public instance of the CERES database donated without the proprietary data. COSMOS DB v2 contained enhancements to the underlying architecture and additional quality review, resulting in 81,602 CMS-IDs with 44,773 unique structures (after removing duplicates). We briefly summarise the database technology and content below.

#### Architecture

**Fig. 2:**
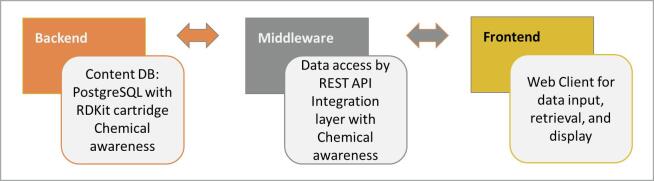
Simplified COSMOS System Architecture: 3-tier system.

COSMOS DB is based on a 3-tiered architecture: client (front end), middleware, and backend. COSMOS DB v2 uses PostgreSQL (v9.3) [28] as the database back-end with the RDKit [29] library and cartridge (RDKit 2014.03.1 with Python 2.7) along with the MOSES2 library (from MN-AM chemoinformatics package). The RDKit cartridge and RDKit/MOSES2 libraries handle chemoinformatics needs for searching and retrieval. Fig. 2 illustrates the high-level conceptual diagram of the COSMOS DB architecture.

One integral role of the middleware layer is the facilitation of the efficient transfer of information between the frontend (for clients) and the backend. This process is implemented using a REST API (Representational State Transfer Application Programming Interface), a common software paradigm for enabling web-based communications.

#### Summary of data model

**Fig. 3:**
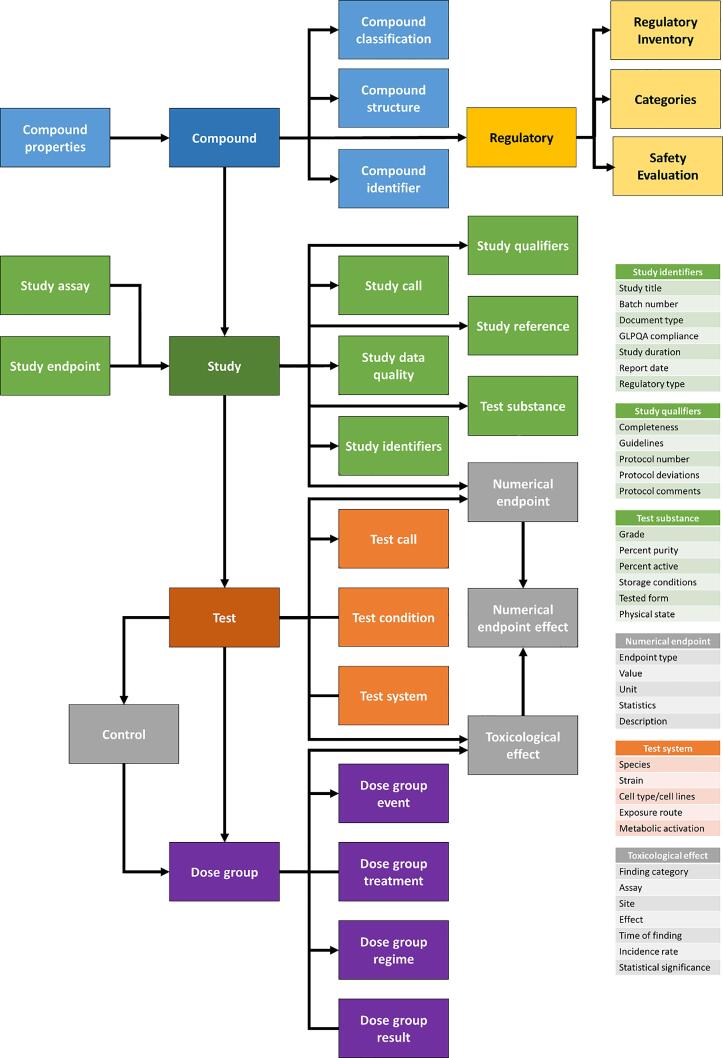
Conceptual Representation of the COSMOS Database.

Fig. 3 shows the high-level logical relationships across various objects: chemistry [structure, compound annotation, identifiers, e.g., CMS-IDs, CAS RNs (Registry Numbers), DSSTOX [30], [31], and other Database IDs, and source names; regulatory information and safety assessment, e.g., Hazard Index or Acceptable Daily Intake, PAFA chemical assessment; toxicity studies, e.g., *in vivo* and *in vitro* endpoints. Whilst the CERES and COSMOS database contents were modelled after ToxML standards [10], the safety assessment section and detailed dose-level toxicity model have been expanded during the COSMOS Project to facilitate ontology-driven data-mining.

***Chemistry Sources*** The COSMOS DB chemistry content was compiled through consolidation of multiple regulatory databases and datasets. Data sources include the public components of the following: FDA CFSAN CERES project [7]; EPA databases (ToxRefDB [18], DSSTox [30], [31], ACToR [32], IRIS [33], and Tox21 inventory [34]); the CosIng [21] Database from the EU containing SCCS opinions link; ECHA REACH Registered Substances Database [35]; US Cosmetic Ingredient Review [36]; US NIEHS NTP [37]; and WHO IARC [38]. Special emphasis was put on the cosmetics-related chemicals, which comprise a major subset of COSMOS DB, i.e., the Cosmetics Inventory. Through CERES public data, food packaging substances were also included in COSMOS DB.

***Chemistry Data Model*** COSMOS DB is substance-centric and the primary identifier, CMS-ID, represents a unique compound, which is equivalent to “substance” as stipulated in other regulatory sources. This identifier was consistent with the CERES database identifier, CRS-ID, for publicly sharable compounds. The chemistry information consists of objects containing compound summary (chemical structure representations), names, registry numbers, and identifiers. A compound (or test-substance) can be either a single substance or a chemical mixture composition, which may consist of one or more structures. Hence, a compound may be composed of multiple structures and a single structure can appear in multiple compounds (many-to-many relationship). A compound is annotated by several attributes: the existence or lack of a connection table (structure representation), stereochemistry and/or double-bond geometry, composition or materials type. The “Related Compound” concept was also implemented to represent multiple forms including parents, ionic species, or metabolites. When a material has an ill- or un-defined molecular formula, a specific “Representative Structure” was defined to provide a chemical structure. For example, alcohol ethoxylated polymeric surfactants were represented with average values of the distributions of alkyl chain length and ethoxylation (EO length). Many of the polymeric structures can be more accurately represented as Markush [39] type of structures; however, for practical reasons associated with the database technology in COSMOS DB, polymeric units were represented by using monomer forms.

***Regulatory Data Model*** As shown in Fig. 3, the regulatory section of COSMOS DB consists of registration inventories, compound categories (substance use type, product category, compound classes, etc.), and safety evaluation results from various regulatory agencies and bodies. Opinions from SCCS [22], human health assessment information on a chemical substance from US EPA IRIS [33], and information from PAFA [8] are the results of safety assessment programs of SCCS, US EPA and US FDA, respectively. The data model for the safety evaluation section allows delivery of quantitative risk measures, e.g., Margin of Safety (MOS), Reference Dose (RfD), or Acceptable Daily Intake (ADI), for a chemical substance with a critical study and effects based on the No Adverse Effects Level (NOAEL). Along with assessment results, the year and the owner of the NOAEL decision and quantitative measure are included. The presentation summarises the assessment results from the regulatory agencies and provides a very convenient way to document and visualise the history and status of the decisions.

***Toxicity Data Model*** The COSMOS data model for toxicity studies has its origin in the CERES Database, which followed the ToxML standard [10] as well as OHT (OECD Harmonised Template) [13] whenever possible. Studies are organised hierarchically as study → test → dose-level objects. Study designs and conditions are included in the data model at the study and test levels. Through the COSMOS project, the dose level information was structured such that various findings from experimental results could be well expressed by an ontology-driven controlled vocabulary. High level structure and relationship of the toxicity database are represented in Fig. 3.

#### Chemoinformatics foundation

COSMOS DB and COSMOS NG were built on PostgreSQL [28] (version 9.3 for v2 and NG). Structure handling and rendering for chemoinformatics databases were developed using RDKit [29]. The RDKit database cartridge was also utilised for efficient substructure and similarity searching employing RD Kit topological fingerprints [40] (RDKit 2014.03.1 with Python 2.7). These dynamically generated fingerprints were selected as the first step in our searching method because these molecular graphics-driven fingerprints can be calculated for all chemicals with structures, unlike several database keys. Regardless of the size of the bit set, any pre-defined features including database keys will not be able to cover all chemicals due to the limitation of matching pre-defined features comprehensively.

The similarity of a pair of structures is calculated by applying the Tanimoto coefficient [41]. The recommended setting for the similarity threshold of the fingerprints used in COSMOS DB is 0.7, whilst the lowest similarity searching is limited to the Tanimoto coefficient of 0.65. For more details, a full description and comparative analysis of similarity measures are available in a recent publication [42].

### Structure curation

#### Initial structure compilation

The structures in the US FDA CERES database have been curated from the Chemical Abstracts Service (CAS) of the American Chemical Society (ACS) for specific internal use for the FDA. Hence, it was necessary to compile connection tables (structures) for the entire COSMOS database from public sources. The two largest structure source collaborators for COSMOS DB were US EPA DSSTox [30], [31] and Procter & Gamble Company, accompanied by curation efforts of the COSMOS partners. COSMOS DB retained the CAS RNs if they were contained in public releases from the regulatory agencies. For example, the CAS RNs from SCCS/CosIng Database [21], ECHA Registered Substances Database [35], US FDA CFSAN CERES [7] and PAFA database [8], US EPA DSSTox [30], [31], ACToR [32], and NTP [37] databases were retained.

#### Structure quality control & quality assurance

After merging and fusing structures and chemical data from all sources, extensive structure quality control (QC) work was performed. The structures were systematically confirmed to have removed duplicates, incorrect representations, or simply wrong records. Quality scores (between 0 and 100) were assigned depending on the completeness of information and the reliability of original structure source. Duplicate detections for chemicals with connection tables were performed by direct comparison of the InChI keys (IUPAC). The structure-compound associations were verified through a set of representations (SMILES, InChI code / keys, 2D CTABs) and identifiers (CAS RNs), and names.

To support the process of structure QC, a structure entry and annotation interface was established so that the COSMOS work group could access the web-based centralised system and the QC forms as part of tools in the COSMOS DB. This activity became the foundation of the COSMOS ID registration. The QC groups included teams from US FDA CFSAN CERES, US EPA DSSTox, and the COSMOS project. The QC review of these structures was focused on conflicts between DSSTox and other sources, and compiled results were reflected in the structure curation process. Based on this learning, a formal quality assurance (QA) activity for review of chemical structures was conducted before the release in 2015. By random sampling of structures in the COSMOS v1 database, records for 442 structures (1%) were analyzed. The approximate percentage of inaccurate structures in production v1.0 of the database was 4.3% if stereo chemistry is ignored and 9.7% when stereo chemistry was considered. Approximately 2.2% of the names may contain errors and 0.5% of the records may have incorrect registry numbers. The error rates corresponding to these elements are provided in the Table 1. Note that similar error rates associated with chemical structure curation efforts has been reported in association with the DSSTox database effort [30], [31].Table 2.

**Table 1:** QA statistics of COSMOS DB v.1 chemistry content.

Elements in Review		# Corrected Records	Error rate (%)
Connection Table		43	9.7
	Connectivity	16	3.6
	Stereochemistry	24	5.4
	Protonation State	3	0.7
Name		10	2.2
CAS Registry Number		2	0.45

**Table 2:** Cosmetics Inventory V1 based on CMS ID & INCI Names.

Source	CosIng REFNUM	INCI Name	CAS + INCI
EU CosIng Database	19,301	19,300	19,473
US PCPC	None	3,575	3,463
Overlap CosIng / PCPC*			3,339
Total Inventory		19,397	

*The PCPC part of the COSMOS Cosmetics Inventory included a list of substances from a book by Bailey [43].

### Cosmetics Inventory

#### Cosmetics Inventory in COSMOS DB

To define cosmetics-related chemicals, i.e., intentional cosmetics ingredients and other substances used in cosmetics products, the Cosmetics Inventory was integrated as a superset of the COSMOS database. It was compiled by fusing EU and US chemistry data sources of the COSMOS DB, namely: the EU CosIng Database [21] and the US Personal Care Products Council (PCPC) list [43] in v1, and then the addition of the US Cosmetic Ingredient Review (CIR) list [36] in v2.

For COSMOS Cosmetics Inventory v1, the CosIng database was queried in April 2011 from the European Union CosIng database website [21]. The inventory file was processed by two indexes, namely, the CAS RNs and INCI [44] (International Nomenclature Cosmetic Ingredient) names. There were 9286 unique CAS RNs, 19,397 unique INCI names, and 66 unique chemical functions in the CosIng inventory. The US PCPC inventory was compiled based on a book published from PCPC containing a list of cosmetics ingredients available in US market [43]. After curating the content, the inventory lists were defined for 3716 unique CAS RNs and 3575 unique INCI names.

Due to the nature of cosmetics and cosmetics-related chemicals, the inventory comprises a large number of botanicals, extracts, mixtures, and polymeric compounds, which made the registration as well as the validation of identifiers and structures particularly demanding. The two inventories (CosIng and PCPC or CIR) were combined by INCI names and CAS RNs as a paired representation (INCI_CAS). Due to the non-unique, many-to-many relationships between the two inventory sources, detection of duplicate structures in the overlaps were performed using InChI keys, wherever possible. For many records without structures, text mining techniques to establish a key word list for controlled names was adopted such that a key word searching (e.g., concatenated INCI_CAS representations) could be applied to detect the names prior to manual inspections. These inventories also included substance use types, e.g., chemical functions from CosIng and technical effects (e.g., Antimicrobial agent, Antioxidant, Colour or colouring adjunct, etc.) from PAFA, which give valuable information pertaining to where the substances had been registered. This COSMOS Cosmetics Inventory was used as a reference set for assigning chemicals as being related to cosmetics.

**Fig. 4:**
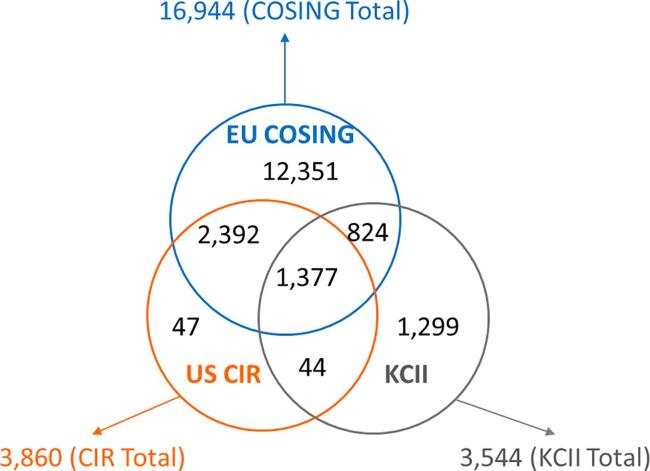
Compound Counts of Cosmetics Inventory (from 3 sources) in COSMOS DB v2 EU CosIng; US CIR; KCII (Korean Cosmetics Institute of Industries).

The Cosmetics Inventory was updated in COSMOS v2 in 2020 with the new CosIng data as well as the US PCPC list. In v2, the chemical inventory and product categories were provided by US CIR and updated based on the list from the FDA’s Voluntary Cosmetics Registration Program (VCRP), with the latter containing over 7700 INCI names [45]. The total CMS-ID contained in the Inventory v1 was 17,100 along with 15,904 INCI names and 9857 CAS RNs. Individual counts and overlaps between EU CosIng and US CIR are shown in Fig. 4.

As depicted in Fig. 4, the COSMOS Cosmetics Inventory v2 also includes a cosmetics inventory from the Korean Cosmetics Institute of Industries (KCII). The addition extends the chemical space of the cosmetics inventories of Europe and USA. The total count of this global cosmetics inventory, with the three inventories combined, consists of 18,334 CMS-IDs, an increase of 6.7% compared to the previous v1 inventory.

#### Characterisation of chemical space of cosmetics Inventory

##### Substance use type

**Fig. 5:**
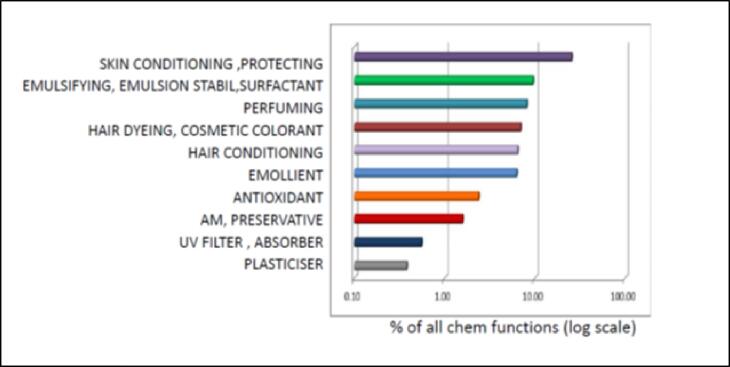
Top 10 Chemical Function in Use of Cosmetics Inventory v1.

Chemical functions in use for substances in the Cosmetics Inventory cover more than 100 chemical function categories. Shown in Fig. 5 are the top 10 use types; we can already find that overlaps between cosmetics and PAFA are primarily due to types of dyes, colourants, antioxidants, antimicrobials, and plasticisers. This inventory has served as a reference set to compare many use types and relevant structural classes.

##### Analysis of structure classes by ToxPrint chemotypes

**Fig. 6:**
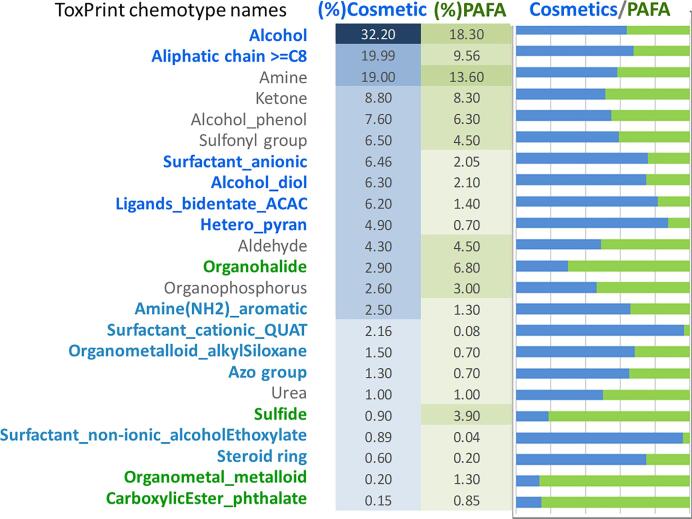
Histogram of Frequency Ratio between Cosmetics Inventory v1 and PAFA database. ToxPrint chemotype names are abbreviated to indicate structural classes in Cosmetics Inventory (blue) and PAFA in COSMOS DB (green). (For interpretation of the references to colour in this figure legend, the reader is referred to the web version of this article.)

The chemical space can be effectively compared between different sources by comparing the frequency histograms for a nominal set of chemotypes representing each set. The cosmetics inventory was again compared with the COSMOS database containing only PAFA contributions from US FDA CFSAN (Fig. 6). Chemotype classes were identified by generating ToxPrint [46] fingerprints from the ChemoTyper [47].

Although COSMOS DB PAFA contains many structurally similar chemicals to those in the Cosmetic Inventory, the strong presence of features characteristic of PAFA database (food additives) is still observed; those features include organohalides, organophosphorus, organometals, phthalates, and sulfides. On the other hand, steroids, surfactants, aromatic amines, pyrans, and alcohol diol groups are found in the inventory enriched with cosmetics-related chemicals.

##### Analysis of structural classes by property space

Due to necessary functions of cosmetics-related chemicals such as skin penetration, hydration/moisture retaining, and emollients, molecular and physicochemical properties of these structures can be quite unique. Properties calculated using CORINA Symphony [48] for both Cosmetics Inventory and COSMOS DB PAFA chemicals included colligative properties and surface activities of molecules: size (molecular weight, molar volume, topological complexity), solubility (water solubility), hydrophilicity/hydrophobicity (logP), polarity, and topological polar surface area (TPSA).

**Fig. 7:**
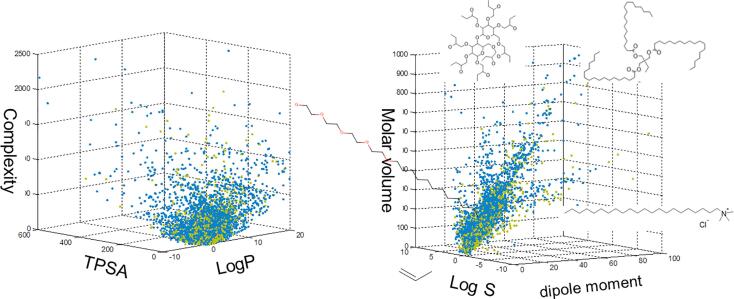
Comparison of Chemical Property Space of Cosmetics Inventory v1 versus PAFA. Cosmetics-Inventory (blue); PAFA in COSMOS DB (green). (For interpretation of the references to colour in this figure legend, the reader is referred to the web version of this article.)

Fig. 7 demonstrates that even similar inventories containing food additives and cosmetics can be distinguished with wide distributions existing in the Cosmetics Inventory represented by this set of simple plots of physicochemical / molecular properties.

### COSMOS DB content: safety evaluation and toxicity database

#### Chemical space of COSMOS DB

COSMOS DB v1.0 was a merged source of US FDA CFSAN (PAFA [8] and CFSAN [7]) and CosIng [21] databases, with additional toxicity data from SCCS [22], EFSA [49] and NTP [37], as well as the safety assessment data from PAFA [8], SCCS [22], and US EPA IRIS [33]. For chemical space comparisons, two large structure sources were considered, namely Cosmetics Inventory and Tox21 [34]. The Tox21 chemical inventory allows for comparisons to a more diverse set of industrial and environmental chemicals [50].

**Fig. 8:**
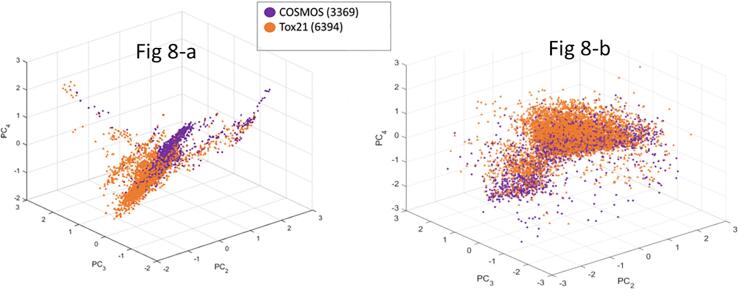
Principal Component Projections of Cosmetics Inventory (3369) Compared to Tox21 (6394). (a) PCA using ToxPrint Chemotypes (present in more than four structures); Fig. 8-b: PCA using CORINA Symphony descriptors.

In Fig. 8, the principal component (PC) projections exhibited clear separations of Tox21 and Cosmetics Inventory structures when employing ToxPrint chemotypes (structural features) and molecular properties from CORINA Symphony. Properties include: molecular weight, number of H-bond donors, number of H-bond acceptors, XlogP, TPSA, polarizability, dipole moment, logS, Lipinski rule-of-five violations, complexity, and diameter. Principal component PC2 in Fig. 8a separates a large percentage of the chemicals in the Tox21 and Cosmetics Inventory. A large cluster of Tox21 chemicals loaded on PC2 is clearly isolated from the cosmetics. Cosmetics chemicals also are differentiated from Tox21 chemicals along the PC3 and PC4 (ToxPrint) axes, as well as PC2 and PC4 (properties) axes. An interesting separation of clusters in property space was observed based on hydrophobicity. The structures found with negative loadings in the PC2 in Fig. 8b represent higher hydrophobicity (higher logP, lower water solubility, lower TPSA, and lower H-bond donors) than the ones in the larger cluster with positive loading in PC2. Also noticed is that both Tox21 and Cosmetics Inventory show separation of the hydrophobic from hydrophilic clusters. Tox21 structures seem to have higher loading in the PC4 axis. This analysis increases confidence that these structural fragments and properties are capable of differentiating chemical space for cosmetics, pesticides, drugs, and industrial chemicals.

#### Safety evaluation data

The “Safety Evaluation” browser in the COSMOS DB offers access to study records from the results of safety evaluation programs across various sources specifying the critical study and associated effects (see Table 3). The “Safety Assessment Database” also contains studies that were considered and evaluated under various regulatory assessment programmes (e.g., US EPA IRIS, SCCS, EFSA) in addition to the critical study. The data model was similar to that used in the PAFA database except that the numerical endpoints were NO(A)EL/LO(A)EL. In COSMOS DB v2, the safety evaluation records span a total of 1065 compounds, of which 1011 compounds (1383 studies) had studies leading to 1003 NO(A)ELs and 884 LO(A)ELs.

**Table 3:** Information Sources for the Safety Evaluation of Cosmetics-Related Chemicals.

Source	Quantitative RISK type & Numeric Endpoints	# with Values	# total Compounds
PAFA	JECFA ADI^ǂ^	200	7202
SCCS	Margin of Safety (MOS) based on NO(A)EL evaluation results	74	135
	NO(A)EL/LO(A)EL in the safety evaluation results	78	133
	NO(A)EL/LO(A)EL in the safety assessment database	140/125	153
IRIS	Oral Reference Dose (RfD) based on NO(A)EL in the safety evaluation results	12	12
	NO(A)EL/LO(A)EL (or BMD/BMDL)* in the safety assessment database	247/242	247
COSMOS/ILSI TTC** Group	NOAEL	552	552

^ǂ^ADIs determined by JECFA were included in the PAFA chemical safety information, separate from the toxicity database.

* Benchmark dose (BMD) and benchmark dose modelling (BMDL).

** International Life Sciences Institute – Threshold of Toxicological Concern, https://ilsi.eu/publication/threshold-of-toxicological-concern-ttc/.

##### Regulatory inventory‘

An inventory concept to locate compounds across the international regulatory agencies gives the users ideas as to where they might be able to find data, even if the data for the particular compound is not found within COSMOS NG. Table 4 includes the inventory list that was available in COSMOS DB v2. COSMOS NG has an expanded and updated list.

**Table 4:** List of Regulatory Inventories in COSMOS v2.

Inventory Name	Unique Compound Counts*
US Cosmetics - CIR (US CIR)	4030
EU Cosmetics - CosIng	16,944
US FDA – GRAS	223
US FDA - PRIOR SANCTIONED	12
US EPA – DSSTox	3860
US EPA – ANTIMICROBIAL inventory	106
US EPA - TOXCAST PHASE II	1891
US EPA - TOXCAST PHASE I	305
EU EFSA – OpenFoodTox	3801
EU ECHA – Registered Substances	4463
Korea – KCII	3544
Japan – HESS	696
US Cosmetics – PCPC**	4030
US FDA – PAFA	7204

*These counts were based on the 2018 update of COSMOS v2. These counts may not represent the numbers from the source.

**This count has been replaced by US CIR list since 2015.

#### Toxicity databases in COSMOS DB

##### US FDA CFSAN databases

***CERES Database*** For more than a decade, US FDA CFSAN has constructed and managed the CERES database containing toxicity studies and regulatory data from various FDA programs. The toxicity data were curated from FDA programs, including Food Contact Substance Notifications (FCN) [51] including food contact substances and impurities, Food Additive Master File (FMF) [52], Food Additive Petition (FAP) [53], Generally Regarded as Safe (GRAS) Notification (GRN) [54], Scientific Committee on GRAS Substances reports (SCOGS) [55], and Specific Prior Sanctioned Food Ingredients [56]. The endpoint with the largest amount of data is genetic toxicity, with the order of endpoints from highest to lowest coverage being Ames mutagenicity, chromosome aberration, mouse lymphoma, and micronucleus studies, followed by 90-day and developmental and reproductive toxicity (DART) studies. Data from CERES had dose-level information and its publicly sharable data were imported to the COSMOS DB.

***PAFA Database*** Strictly speaking, PAFA technically belongs to the genre of safety assessment databases. The purpose of this database is not to give dose-level information of detailed regular toxicity data, but rather to capture numerous quantitative numeric endpoints of highest no effect level (HNEL) and lowest effect level (LEL) values from the various studies to support post-market safety assessment(s). Over 7200 food and color additive related substances are attached to numerous endpoints (27 endpoints) from more than 12,000 studies, including genetic toxicity *in vivo* and *in vitro*, oral toxicity (target organ repeated-dose, reproductive/developmental toxicity), and acute toxicity. Of these studies, more than 3500 oral toxicity studies are available for nearly 1000 test substances. Genetic toxicity studies are available for more than 550 compounds. Of the 7200 compounds in the US FDA CFSAN PAFA [8] database, more than 3000 are also in an inventory called “Substances Added to Food (formerly EAFUS)” [57], which represents substances added directly to food that FDA has either approved as food additives, colour additives or GRAS ingredients; substances approved for specific uses in foods prior to September 6, 1968 (Prior-sanctioned substances); flavouring substances evaluated by FEMA and JECFA; and substances formerly used in food that are now prohibited for use in food; delisted colour additives and substances “no longer FEMA GRAS”.

Although the data were aggregated up to the study level, the database followed strict inclusion criteria and standardised controlled vocabulary, as well as systematic assessment of the study quality specifying whether a study was acceptable to the current FDA standard, met the minimum requirements, or was unacceptable.

##### oRepeatToxDB

In the European Union, some cosmetics ingredients such as UV absorbers and hair dyes are regulated, for which SCCS writes opinions [22]. Although available as opinion documents, the data content was not incorporated into a searchable database. To this end, the COSMOS Project executed a data compilation activity to enrich the cosmetics space with the SCCS data. During this time, toxicity data for 228 compounds were curated from 340 oral toxicity studies by COSMOS partners; the majority of data were from SCCS (1 2 5) and FDA CFSAN (90). There were 186 cosmetics-related chemicals (100 hair dyes from SCC) and 42 impurities from packaging materials (FDA CFSAN). Unlike PAFA, this database provides toxicity effects data at dose level in addition to the aggregated NO(A)EL/LO(A)EL values from a critical study for a given compound. The data were compiled in the ToxRefDB data entry tool adjusted to COSMOS DB to promote the data exchange between the two databases.

**Fig. 9:**
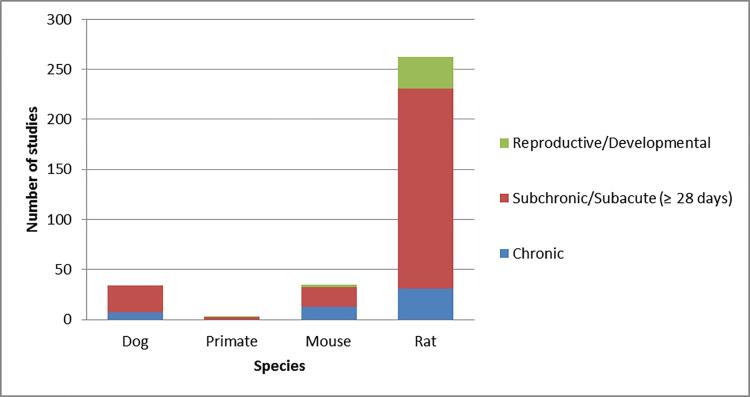
Profile of Studies in oRepeatToxDB for Species and Study Types.

These toxicity studies mainly include rats, mice, dogs, and primates (monkeys). Oral repeated-dose toxicity in rats treated for 90 days are the most common studies, as shown in Fig. 9.

Because it contains a high percentage of cosmetics ingredients such as hair dyes, UV absorbers, and antimicrobials, evaluation of structures associated with the most strongly affected target organs allowed insights into the organ toxicity due to exposure to cosmetics-related ingredients. The order of frequencies of organ toxicities in the database was liver, kidney, stomach/forestomach, and spleen of rats and mice; there were insufficient dog studies to draw statistical conclusions. Based on these data, rat seems to be the species most sensitive to liver effects and generally the most sensitive species to cosmetics-related chemicals in the oRepeatToxDB. Other affected organs are lung, thyroid, skeletal muscle, lymph node, bone marrow, heart, adrenal, pancreas and large intestine.

**Fig. 10:**
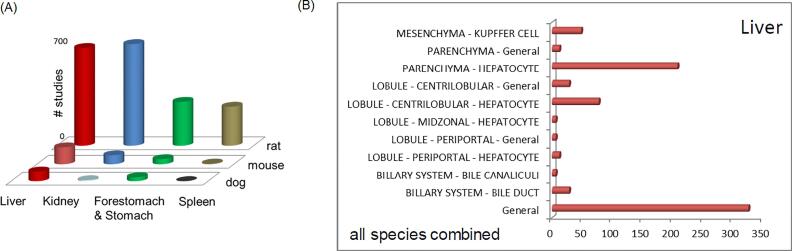
Profile of oRepeatTox-DB against Species, Target Organs and Cells. (A): The most sensitive target organs in COSMOS oRepeatToxDB: liver, kidney, forestomach/stomach and spleen; Fig. 10-B: Phenotypic effects recorded for liver, occuring to be the most sensitive target organ in COSMOS oRepeatToxDB. Overall, 53 pairs of “site-effect” category were found.

The ontology-based controlled vocabulary in COSMOS oRepeatToxDB is designed to relate organs to tissues/segments and, more specifically, to cells, thus enabling mapping phenotypic effects (observed at higher organism levels) to biological processes occurring at the cellular level. The recorded histopathological lesions were grouped by organs and/or systems (Fig. 10A). Overall, 234 phenotypic lesions associated with 36 organs/systems (covering 120 sites of “organ/system-tissue/segment-cell”) are recorded in oRepeatToxDB for 127 compounds. Rat turns out to be the most sensitive species, whereas liver was the most sensitive organ. Fig. 10B presents 53 unique phenotypic liver lesions recorded for seventy chemicals in thirteen sites designated “tissues/segments” and “cells”.

##### REACH IUCLID-6 database

The data from the REACH initiative, made publicly available from the Registered Substances Database [35] of ECHA, have been used in numerous chemical safety assessment activities, including read-across activities. Publicly sharable data from the original substance’s dossier is made available by ECHA in the IUCLID format, e.g., IUCLID-6 [58]. “IUCLID is a software to record, store, maintain and exchange data on intrinsic and hazard properties of chemical substances” [58].

The study data captured in IUCLID-6 were implemented in 2020 for COSMOS NG [24] to promote the role of chemoinformatics in chemical safety assessment within a knowledge hub, for example, read-across activities. For chemistry, the full IUCLID-6 contains 9995 CMS-IDs in total, of which 9641 compounds (7330 structures) are mapped to IUCLID dossiers. Human health-related toxicity endpoints labelled “experimental study” or “key study”, were extracted, data modelled to fit to COSMOS DB, and terminologies standardised using the controlled vocabulary, resulting in 74,815 studies covering 91 endpoints for 6542 compounds. A high-level profile of the data content is listed in Table 5.

**Table 5:** Toxicity Information Profile of IUCLID-6 in COSMOS DB.

Study Types (Aggregated)	Total # of Studies	Total # of CMS ID
Genetox *In vivo*	3423	1988
Genetox *In vitro*	14,963	5423
Repeated Dose Toxicity	7347	3628
Reproductive / Developmental	7022	3050
Skin Sensitisation	6349	4414
Skin Irritation / Corrosion	8144	5265
Dermal Absorption / Permeability	607	441
Toxicokinetics	2122	1040
Acute Toxicity	15,631	5842

The addition of the IUCLID-6 database to COSMOS NG increased the compound counts by 5179 compounds (3324 structures). All of the 3324 structures were curated by MN-AM and LJMU and evaluated by MN-AM for registration in the COSMOS registry. New structures along with the data will increase the utility of the database in chemical safety assessment, and especially in read-across workflow.

In future updates, the COSMOS DB data model will be modified to incorporate environmental studies as well as secondary-sourced information such as read-across, QSAR, and other calculation-based study entries available at the IUCLID API site [59].

### COSMOS DB toxicity data quality assessment

One of the most important tasks of the COSMOS Project was to establish a data governance process [60] to authorise, characterise, and control the use of the qualified data. Special attention was devoted to this issue for endeavours such as COSMOS DB and NG where large amounts of data from many diverse sources are merged, fused, and evaluated before being consumed by users. To this end, the project developed methods and tools to qualify and quantify the data, whenever possible, to assess data quality and reliability, and then finally to control the use of data at a given reliability.

#### COSMOS MINIS criteria

The acceptance of any *in silico* assessment based on prediction models greatly depends on the quality of data used for their development, thus the collection and curation of high-quality data are of major importance. For example, the study inclusion criteria for the oRepeatToxDB were developed to support the construction of the COSMOS TTC [61] dataset as well as structural knowledge development for target organ toxicity. The full description of COSMOS MINIS (MINImum Study) Criteria is provided in Appendix A.

In evaluating data quality in order to judge study reliability, the following perspectives were considered:

1) Does the study design provide enough parameters to be considered “usable” in safety assessment?

2) Are the experimental results supported by the study design?

3) Are the conclusions deemed “reliable/believable/interpretable” by regulators or domain experts?

Of these three perspectives, the first is objective, the second can be made systematic so that the evaluation results are given according to certain rules (algorithms), and the third point is subjective and requires domain experts’ opinions.

For the first two objective criteria, the COSMOS MINIS criteria were developed by Yang et al. to define the minimum inclusion of data for a highly curated toxicity database [61]. The criteria specified minimum acceptance of data for major study design parameters as well as for assessing quality of the results. The minimum criteria for study parameters can be implemented in the database automatically to assist evaluation of data usability. On the other hand, an assessment of the reliability of conclusions needs to include opinions from domain experts. In the COSMOS TTC effort, the latter was addressed by an ILSI Europe Expert Group.

#### COSMOS MINIS Grade

For assigning systematic data quality more quantitatively, the COSMOS MINIS Grade scoring system was devised. The first two objective perspectives were elaborated to five aspects of MINIS criteria to systematically develop a numeric grade. They include:

1) OECD guideline / deviation and GLP (Good Laboratory Practice) compliance;

2) Study design – level1 (e.g., species, strains, cell lines, metabolic activation);

3) Study design – level2 (e.g., concentration / dose levels and ranges, number of duplicates, repeats, assay techniques);

4) Control information (concurrent, types of control, etc.);

5) Results reporting completeness (depending on endpoints).

Appendix A lists the sixteen rules based on MINIS criteria that were coded to define the MINIS Grade after evaluating the five aspects of data quality.

#### Results Interpretation: opinion scores

As mentioned above, the third perspective of study reliability deals with interpretations of the results as addressed by domain experts. Quite often, their opinions are expressed in verbose descriptions whilst representing quantitative measures in very simplistic scores such as Klimisch scores, which implicitly mix the study protocols and results reliability in four categories [62].

Opinion Scores for study interpretation are assigned to one of five groups:

• High (Score = 5): Experimental results are internally consistent and experts agree with the study conclusion.
• Medium High (Score = 4): Experts agree with the study conclusion when the OECD guideline deviations include somewhat less critical factors for experts, e.g., the Ames test system with strains lacking WP2 or TA102 strains while giving negative outcome.
• Medium (Score = 3): Experts may agree with the study conclusion although the guideline deviations include important factors, e.g., use of insufficient number of dose groups or number of animals or unsatisfactory dose separations.
• Medium Low (Score = 2): Experts may not agree with the study conclusion due to questions related to serious guideline deviations of the test system or test design and data on which the conclusion was based, e.g., replicates, control data, and interpretations on findings details.
• Low (Score = 1): Data are highly aggregated as summary data, but conclusion may be usable according to experts.

#### Study Reliability Likelihood

The “Study Reliability Likelihood” is developed as a composite reliability score for toxicity data. The measure is defined based on the data quality (MINIS Grade) and the results interpretation by experts (Opinion Score). The measure is defined as a likelihood such that it can be used within various evidence-based workflows during, for example, a read-across process. These measures therefore needed to be available to users from the database (e.g., for MINIS Grade) and via a workflow tool (e.g., Opinion Scores) to define the study reliability.

Currently in the public domain, ToxRTool [63] is available to determine the Klimisch Score as a study reliability measure. Although the Klimisch score is well established and widely used, it lacks detailed criteria for assigning data quality to scores. The Joint Research Centre (JRC) of the European Commission developed a software-based tool (ToxRTool) to provide comprehensive criteria and guidance for reliability evaluations of toxicological data [64]. It is applicable to various types of experimental data, endpoints and studies (study reports, peer-reviewed publications) and leads to the assignment of data to Klimisch categories.

To systematically enable the process of determination of study reliability, COSMOS NG provides a MINIS Grade for toxicity studies; COSMOS NG also offers a web service tool for assigning the Opinion Score (study interpretation by experts) described in the section 2.5.3. Table 6 lists the data quality and study reliability likelihood for various cases where MINIS Grade and Opinion Scores are given.

**Table 6:** General Study Reliability Likelihood Based on MINIS Grade and Opinion Scores.

Cases	Data Quality (based on MINIS Grade)	MINIS Grade (Section 2.5.2)	Opinion Scores (Section 2.5.3)	Study Reliability Likelihood*
A	Meets all five aspects listed in 2.5.2. Also specific and detailed values (e.g., incident rate, treatment-relatedness per effect or genetic toxicity measure, cytotoxicity, etc.) at a given conc./dose level are available along with the test conditions and control information. (missing none of 16 rules)	5	5 | 4 | 3 | 2 | 1	1 | 0.95 | 0.85 | 0.75 | 0.6
B	• Meets all five aspects listed in 2.5.2, but concentration/dose level detailed effects are explicitly available while assessed data are presented. (missing none of 16 rules) • Study follows OECD equivalent guideline but has either missing records due to being “not specified” or “not-conducted”; it has at least one deficiency in the five aspects (section 2.5.2). Some deviations included when the number of dose groups or number of animals used was not sufficient or dose separation was not satisfactory (missing 1 or 2 out of 16 rules)	4	5 | 4 | 3 | 2 | 1	0.95 | 0.9 | 0.8 | 0.7 | 0.6
C	Studies either missing records or not conducted and at least two deficiencies in the five aspects defined in 2.5.2. (PAFA dose level info missing; missing 3 or 4 out of 16 rules)	3	5 | 4 | 3 | 2 | 1	0.9 | 0.85 | 0.75 | 0.65 | 0.6
D	Studies that are either missing or not conducted and more than two deficiencies in the five aspects. (missing >4 out of 16 rules)	2	5 | 4 | 3 | 2 | 1	0.85 | 0.8 | 0.7 | 0.6 | 0.5
E	Summary Data Only; None of the five in the list are met.	1	5 | 4 | 3 | 2 | 1	0.8;0.75;0.65;0.6;0.5

*Study Reliability Likelihood was modelled based on MINIS Grade (section 2.5.2) and Opinion Scores (section 2.5.3) for each expert opinion at a given MINIS Grade in a scale between 0 and 1. These likelihood values are amenable for probabilistic treatment when used for weight of evidence combinations. The inserted plots depict how Study Reliability Likelihood decreases as Opinion Score decreases for each MINIS Grade.

MINIS Grades define five categories (Appendix 1), ranging from “meeting all 16 pragmatic rules of COSMOS MINIS criteria (GRADE = 5)” to “Summary data only” (GRADE = 1)”. It is then combined with the experts’ opinions to obtain study reliability. The quantitative value of “study reliability likelihood” is defined from 0 (not assessable or not reliable) to 1 (highly reliable). In estimating the study reliability likelihood, the expert opinions were given more weight than the COSMOS MINIS Grade. If any relevant data exist, the lowest reliability value was estimated to be around 50% (0.5 likelihood). The data quality attributes listed in Table 6 are important also for characterising general toxicity studies. This methodology was reported to EFSA for *In Silico* Assessment of Genetic Toxicity Impurities [65].

#### Data quality assessment results

##### QA statistics of oRepeatToxDB

The oRepeatToxDB Data were evaluated using a systematic quality assurance (QA) process before COSMOS v1 was released. The results of the quality assurance (QA) on oRepeatToxDB using MINIS criteria resulted in 0.57% erroneous records (e.g., mistakes in animal counts or incorrectly inserted effects) and 5.2% missing records (e.g., effect descriptions). This exercise provided insight into where errors occur even under a strict database construction process.

##### Applying the MINIS Grade to COSMOS DB v2

**Fig. 11:**
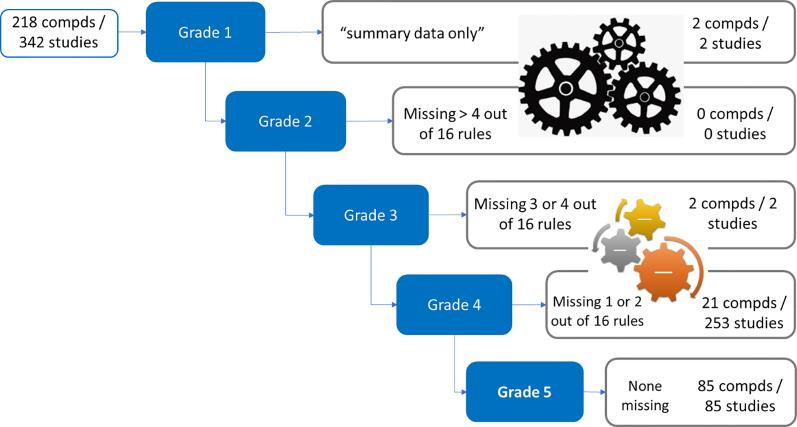
Process of Assigning MINIS Grade. Descriptions of MINIS Grades can be found in Table 6.

Quite often during the *in silico* chemical safety assessment process, there is a need to assess the study reliability such that the uncertainty of the final outcome can be judged. To assure data quality, the COSMOS project established a robust data quality evaluation process through application of the COSMOS MINIS criteria and Reliability Score, as shown in Table 6. In addition, since multiple sources of data are considered during this process, it is also desirable to be able to compare data from different sources consistently and systematically. Fig. 11 depicts the steps applied to COSMOS DB to algorithmically determine the MINIS Grade.

**Fig. 12:**
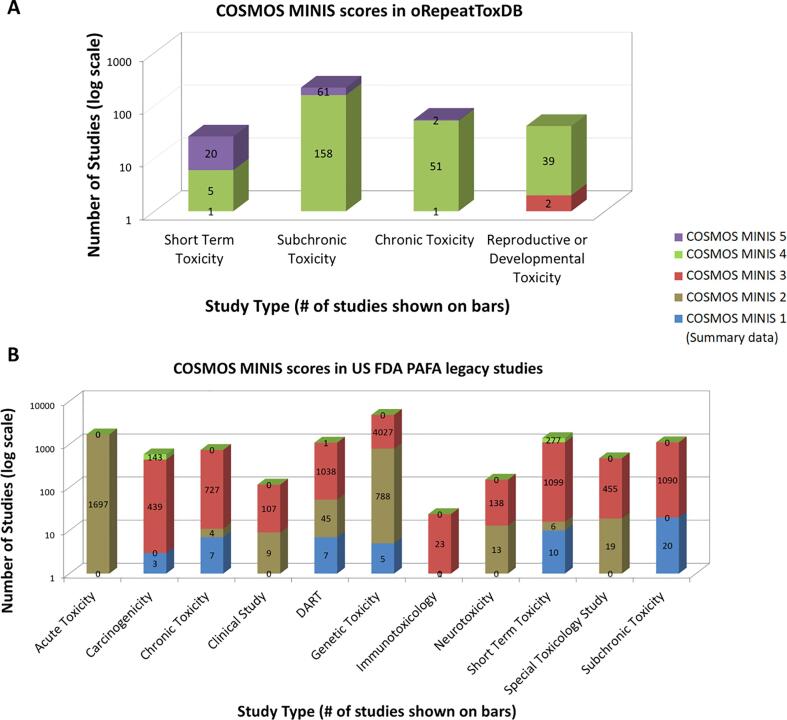
Results of Applying the MINIS Grade to COSMOS DB.

The logical rules of MINIS Grade (in Table 6 and Appendix 1) were coded into a decision tree, which then was applied to oral toxicity data in COSMOS DB. As shown in Fig. 12, most studies in oRepeatToxDB are MINIS Grade 4, i.e., providing acceptable details of studies. For the PAFA database, a large fraction of studies is assigned to Grade 2 and 3 due to PAFA studies having no dose-level information.

Making use of this MINIS Grade combined with the Opinion Score for estimation of study reliability likelihood was presented in Table 6. This final study reliability value, given as a likelihood, can further support the reliability of NOAEL/LOAEL values important in *in silico* predictions and read-across.

## The potential role of COSMOS NG within a knowledge hub

### COSMOS NG and knowledge hub architecture

The new COSMOS NG extends the COSMOS DB to include *in silico* tools and external workflows. The access and integration layer has been updated such that the system can function as a public knowledge hub where users can share data, knowledge, and results of analysis. In the COSMOS NG, a new version of PostgreSQL [28] (v.10) is used along with the RDKit [29] library and chemistry cartridge (2018.09.1, Python 3.6), and MOSES 3 libraries. For similarity searching, although RDKit Topological Fingerprints [40] were still used, additional useful fingerprints such as ToxPrints [46] and others were added. The REST API middle layer was updated to prepare for handling multiple external sources and to function as a public knowledge hub for *in silico* chemical safety assessment in the future.

**Fig. 13:**
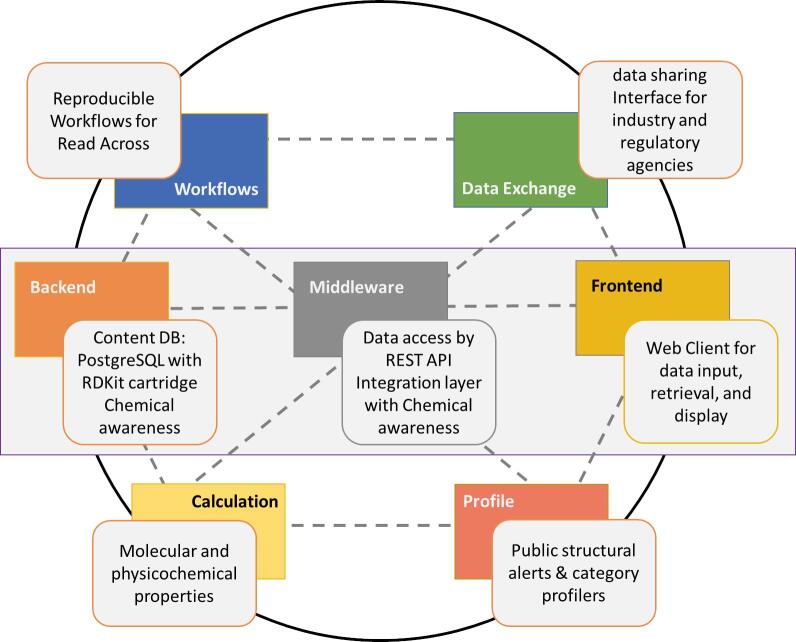
High Level Conceptual Architecture for COSMOS NG as a Core Component for a Knowledge Hub.

Comparing the high-level conceptual view of the COSMOS DB v2 architecture in Fig. 2 to that of COSMOS NG in Fig. 13, the latter offers more tools, including “Calculation”, “Profile”, “Data Exchange/Sharing”, and “Workflows”. This architecture would become part of the core foundation of the COSMOS Knowledge Hub.

### Chemical similarity and profiling of structures

#### Structure-Based similarity

For general similarity searching from the query page, COSMOS NG uses RDKit topological fingerprints within the RDKit [29] cartridge available in the PostgreSQL [28] database. The COSMOS NG also provides ability to use more than one fingerprints in comparing the similarity of structures.

Within the data table for manipulating the retrieval hit list, COSMOS NG provides other structural fingerprints such as ToxPrints [46] and MACCS database keys (public) [89]. For all fingerprints, Tanimoto coefficients are calculated for each structure in the hit list against a target structure to estimate the structure-based similarity. The results can be sorted on similarity based on any selected fingerprint. The importance of examining structures using different types of fingerprints (e.g., dynamic generation or pre-defined expert features) was articulated in detail in previous publications [42], [90].

#### Property-Based similarity

As shown in Fig. 8-b, chemical space analysis using both molecular and physicochemical properties, similar chemicals can be profiled by a group of properties dictating chemical reactivity or affinities toward protein molecules. To enable this within COSMOS NG using the features shared with public from ChemTunes•ToxGPS® [91], molecular and physicochemical properties can be calculated, for example, using CORINA Symphony Community Edition [92]. Based on the selected properties, property-based similarity can be derived from a Pearson Correlation Coefficient or Euclidean Distance. Pearson correlation gives the covariance of the two variables divided by the product of their standard deviations. Since these values vary from −1 to 1, the quantity is rescaled to range from 0 to 1 to be used as a measure for the property-based similarity. The Pearson similarity is then calculated simply by eq (1):

(1) PearsonSimilarity=(1+r)/2

where r is the conventional Pearson Correlation Coefficient. Details on the Pearson Similarity can be found in a previous publication [42]. For property-based similarity measure based on Euclidean distance, the Euclidean distance between each pair of structures is calculated in property space, using standardised property values. We then use a common method for calculating a similarity measure, scaled from 0 to 1, from a standardised Euclidean distance by eq (2):

(2) EuclideanSimilarity=1/(1+StandardisedEuclidean Distance).

Pearson similarity works well when similarity is based on the extent to which properties are correlated, whereas the Euclidean similarity is preferred when similarity is based on the extent to which the properties are similar in value.

To illustrate an example, a simple set of structures similar to propyl paraben is queried in COSMOS NG. After searching for similar structures with RDKit similarity greater than 80%, four structures (1 target and 3 analogue candidates) were selected to compare both structure- and property-based similarity.

In selecting properties to evaluate the similarity of the four structures (methyl, ethyl, propyl and butyl parabens), properties that would be more sensitive to variations in alkyl chain lengths were calculated, including number of rotational bonds and logP along with molecular weight, and complexity. Table 7 lists the data exported from COSMOS NG for the four parabens with structure-based similarities and selected molecular properties. Similarity measures can then be calculated using either the Pearson or Euclidean similarity formula defined in section 3.2.2.

**Table 7:** Exported Data Table Containing Structure Similarity and Property Values.

CMS ID	Name	Fingerprints (Tanimoto Coefficients)				Calculated Properties				
		MACCS	RDKit Mol Fingerprint	ToxPrint Fingerprint	Liver BioPath	# Rotational Bonds	Molecular Weight	Complexity	TPSA	XlogP
CMS-2411	Propyl paraben	1.00	1.00	1.00	1.00	4	180.2	159.6	46.5	2.32
CMS-216	Butyl paraben	0.94	0.94	0.91	0.90	5	194.2	171.4	46.5	2.87
CMS-2412	Ethyl paraben	0.93	0.92	0.80	0.67	3	166.2	147.9	46.5	1.98
CMS-2413	Methyl paraben	0.81	0.82	0.70	0.50	2	152.1	136.3	46.5	1.55

Molecular weight (g/mol); TPSA (Å^3^); no units for # of rotational bonds, complexity, and XlogP.

**Fig. 14:**
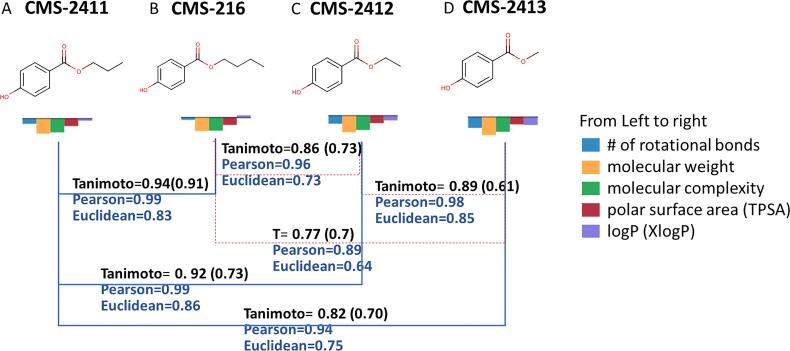
Property Similarity Comparison for Potential Analogues of Parabens. A: Propyl paraben (94-13-3); B: Butyl paraben (94-26-8); C: Ethyl paraben (120-47-8); D: Methyl paraben (99-76-3). Tanimoto coefficient values in parenthesis are from ToxPrints.

In Fig. 14, the four structures were compared using structure and property-based similarities for all pairs. Within the constraints of the various small data sets in this study, Euclidean similarity seemed better at differentiating the potential analogues than the Pearson similarity. It was also better correlated with the structural similarities given by ToxPrints or RDKit fingerprints.

The above example illustrates how selected properties can be used to further differentiate similarities in combination with structural fingerprints. When the combination of both structure and property-based similarities is desired, an Analogue Quality reflecting both measures can be calculated by eq (3) [42]:

(3) $$\text{Analogue}\;\;Q\text{uality}=\;\sqrt[{N}]{\prod_{i=1}^{N}{\left(SimilarityMeasure\right)}_{i}}$$

Similarity measures can be obtained from both structure- and property-based parameters; in addition, more than one attribute for each type is allowed [42]. For example, both physicochemical properties and assay results can be used for property-based similarity measures in a way similar to obtaining structure-based similarity by using different fingerprints (e.g., ToxPrint and RDKit). Based on the analogue quality, compounds B, C, and D would be judged reliable analogues of Compound A in the absence of experimental data. Determining the combined analogue quality will be discussed with the case study below.

Chemical similarity based on biological activities has been applied to Adverse Outcome Pathway (AOP) and high-throughput screening (HTS) assays; PK/TK data as well as toxicogenomics or transcriptomics data can also be utilised as parameters for similarity profiling method. Sets of profiles such as these will become essential in next generation risk assessment (NGRA) [26], [27] and within the general new approach methodology (NAM) [93] paradigm in regulatory assessment. The new features in COSMOS NG help users prepare the information necessary for an assessment based on query results and data obtained by applying the shared tools from ChemTunes•ToxGPS® [91].

#### Chemical category

One of the new additions to the COSMOS NG is the ability to profile and group compounds by categories and pathways. Well-known chemical categories or mode-of-action (MoA) chemotypes are available for mutagenesis [66], genotoxic carcinogens [67], [68], DNA binders [69], [70], [71], protein binders [72], [73], [74], [75], [76], [77], [78], [79], liver toxicity [80], [81], [82], [83], [84], [85], [86], [87] and DART structural rules [88]. If the structure matches any of the categories defined by chemotype fragment, the structure will be associated with particular categories or rules. Examples of chemotype rules highlighted within a structure are described in the Section 3.3.2 (Step-4).

**Fig. 15:**
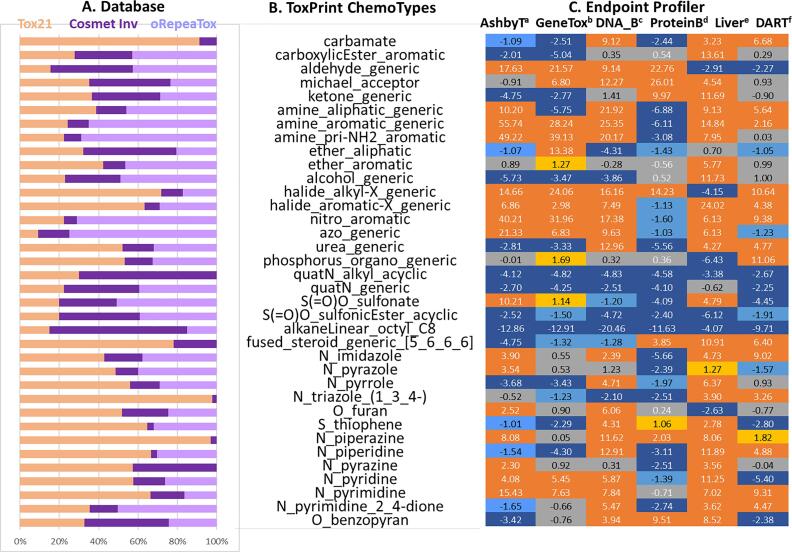
Characterization of the combined databases for endpoint profilers per chemotypes. Col A: a histogram of relative frequencies of each chemotype in Cosmetics Inventory (purple), oRepeatToxDB (lavender) and Tox21 (pale orange); Col B: selected chemotypes matched in the combined database (chemotype names are abbreviated to indicate structural classes); Col C: z-scores of each profiler per ToxPrint chemotype (dark orange: z ≥ 2; orange: 1 ≤ z < 2; gray: −1 < z ≤ 1; light blue: −2 < z ≤ −1; dark blue: z ≤ −2). (For interpretation of the references to color in this figure legend, the reader is referred to the web version of this article.)

**Fig. 16:**
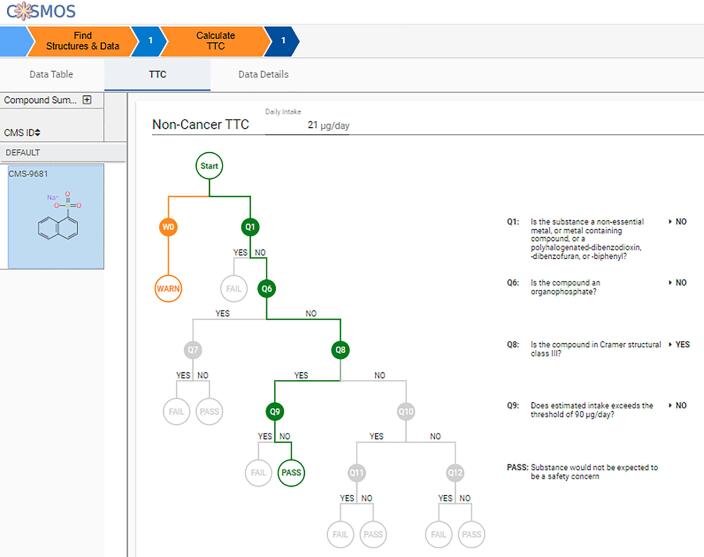
TTC Tree from the COSMOS NG Workflow.

Based on these structural groupings, Fig. 15 illustrates results of further analysis based on selected ToxPrint chemotypes and endpoint profilers against the combined 9863 structures from the three databases, i.e., Tox21, the COSMOS Cosmetics Inventory and oRepeatToxDB. Column A shows the relative frequencies of each ToxPrint chemotype (in Col B) in three databases. Column C represents the endpoint profiles against the selected ToxPrint chemotypes for the full database. For a given endpoint profile, z-scores are calculated and colour-coded from orange (positive association) to blue (negative association) Fig. 16.

The ToxPrint chemotype classes of aromatic amine, nitro, and azo groups were more prevalent in oRepeatToxDB than others due to the focus on hair dyes, some of which were associated with genetic toxicity and genotoxic carcinogenicity. On the other hand, the Cosmetics Inventory included a high proportion of compounds containing quaternary alkylammonium groups and alkyl chains longer than 8 carbons, structures that exhibited mostly negative association with the six endpoint profilers. For Tox21, carbamates, aromatic halides, urea, steroids, and several heterocyclic nitrogens are associated positively with liver or developmental/reproductive toxicities. Profiling compounds is the first step in hypothesis formation in chemical safety/risk assessment.

### Guided workflow for threshold of toxicological Concern (TTC)

COSMOS NG offers an improved version of a pragmatic risk assessment tool for assessing the Threshold of Toxicological Concern, a feature shared by ChemTunes•ToxGPS®. Published TTC datasets are still downloadable from the “downloads” link in the COSMOS NG dashboard. As in COSMOS DB, the new version employs the Cramer classifications [94] implemented in Toxtree [95] for interoperability and consistency. The new implementation generates warnings to notify the user when Toxtree is known to conflict with other implementations [61].

To illustrate with an example, we use a simple food contact substance (sodium 1-naphthalenesulfonate), whose cumulative daily intake is reported to be 0.35 μg/kg-bw/person/day [97]. By running the TTC workflow, we will confirm that the use of this chemical at the CEDI level is not expected to cause safety risks.

Executing the non-cancer TTC decision tree [96], the system assigns Cramer Class III for sodium 1-naphthalenesulfonate. A warning message notes that the Toxtree assignment for this compound type has been reported to conflict with other tools [61]. The TTC tree then checks whether the query compound belongs to the “cohort of concern (COC)” that should not be waived by TTC approach. If the query compounds are from one of the five groups (metal, metal containing compounds, polyhalogenated dibenzodioxin, dibenzofuran, and biphenyls), the tree stops while firing a “Fail” message (Q1). Sodium 1-naphthalenesulfonate does not belong to the COC group, nor is it an organophosphate (Q6), but was classified as Cramer Class III (Q9). Since the daily intake of 21 μg/day/person is lower than the Class III threshold of 90 μg/day, the conclusion of “Pass” is returned, meaning that the use of this food contact substance in oral application is not expected to cause an appreciable safety concern.

### Guided workflow for read-across

To illustrate the chemical safety assessment workflow using COSMOS NG and its tools, this section presents a simple read-across case study for hair dyes. The key features of COSMOS NG include components shared by ChemTunes•ToxGPS® [91]. Although many of these steps and calculations are executed automatically in the commercial ChemTunes•ToxGPS®, public COSMOS NG users are able to follow the workflows by exporting various Excel sheets where analysis can be supported. The Supplementary Information provides all results of this guided workflow in Excel worksheets with embedded formulae for executing calculations.

In the next update of COSMOS NG, web services will include external QSAR models such as VEGA [98] and COSMOS PBK simulations [5] for several case study structures.

#### Steps to identify analogue candidates

A target compound of HC RED NO. 7 (CMS-23938; CAS RN 24905-87-1), a hair dying agent, is considered for the evaluation of systemic toxicity. Analogue candidates of this hair dye were compiled from COSMOS NG, which were selected by structure/property similarity and will be qualified as potential analogues according to the steps below.

**Fig. 17:**
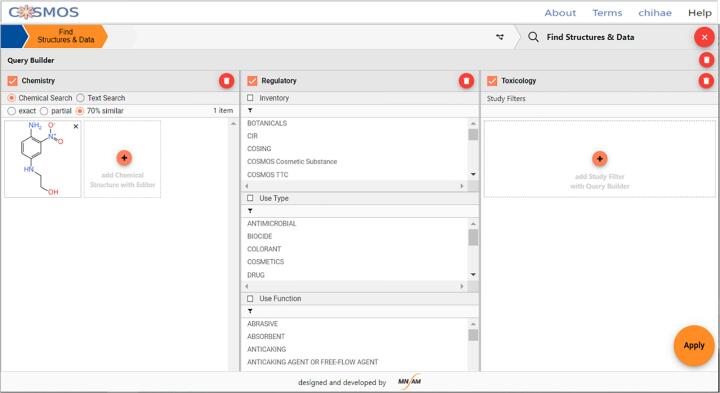
Screenshot of COSMOS NG Query Builder.
  o Sort structures in the order of similarity measure (default: RDKit Topological).
  o Select similar structures containing the core substructure of the target.
  o Note the availability of tox data in the database in the “Data Summary” column.

• **Step 1: Similarity Searching & Selection of Similar Structures**
  o Select structure search for similarity greater than 70% in the database.
  o Enter query structure (method; either drag and drop the mol or SMILES file or by drawing in the JSME molecule editor) to the query builder (Fig. 17)

At this point, 11 compounds with toxicity data are retrieved from the database at the similarity threshold of 70% by RDKit topological fingerprints (in RDKit chemistry cartridge). The “Data Table” view provides the hit list detailing the compound information, availability of toxicity data, and Tanimoto coefficients for various fingerprints implemented in the public COSMOS NG. Similar structures are identified by sorting the records using the Tanimoto coefficients of selected fingerprints, while keeping structures with the core substructure (1,4-amino-2-nitrobenzene), and then keeping the structures having systemic toxicity data. Three structures (CMS-43204, CMS- 60520, CMS-72054) were identified for the target of HC Red No. 7 in COSMOS NG as similar structures.

**Fig. 18:**
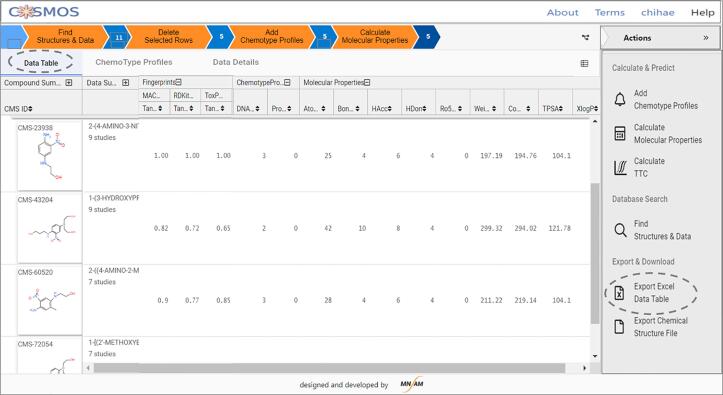
Screenshot of COSMOS NG Data Table.
  o Calculate properties to be included in the data table as shown in Fig. 18.

• **Step 2: Calculate Properties Within COSMOS NG Data Table**
  o Select properties that can provide insights and bring out the distinction between similar structures. For example, Fig. 18 lists nine whole molecule properties that were calculated for the four structures.

In general, the selection of properties would depend on the toxicity findings in question. For example, if toxicity endpoints are known to be driven by chemical reactivity, such as skin sensitisation or genetic toxicity, properties indicating such reactivity would be useful. They are usually calculated by quantum mechanical descriptors of heats of formation and HOMO/LUMO properties, which are only available in the ChemTunes•ToxGPS®. When using whole molecule properties from the public set in COSMOS NG, we recommend rotational bonds, hydrogen bond acceptors and donors, molecular complexity, and logP. For toxicity endpoints where receptor binding is important, shape descriptors (in commercial system only) would also be relevant. In this case, rotational bonds and polarizability can be used in COSMOS NG. When water solubility or melting points are important, physicochemical properties available from the EPA CompTox Dashboard in addition to the CORINA Symphony molecular properties can be used. Fig. 18 displays a screenshot from COSMOS NG after fingerprints (structure-based similarity), molecular properties (property-based similarity) and chemotype profilers (mode action groups) have been calculated.

• **Step 3: Export the Data Table and Analysis to Determine Structure-, Property-Based Similarities and Analogue Quality**
  o Export structure-based similarity and molecular properties in the Data Table as Excel Sheet from COSMOS NG (Fig. 18).
  o Calculate the similarity measures based on fingerprints and properties following the same process as in Table 7, Table 8 of the previous section.

**Table 8:** Calculation of Pearson and Euclidean Similarities based on Property Values.

CMS ID		Standardised Properties*					Property-Based Similarity			Fingerprints-Based		Analogue Quality (AQ)	
		# Rotational Bonds	Weight	Complexity	TPSA	XlogP	Euclidean distance	Euclidean similarity	Pearson similarity	ToxPrint	RDKit	AQ (Pearson)	AQ (Euclidean)
CMS-2411	Propyl	−0.19	−0.56	−0.51	−0.27	−0.08	0.00	1.00	1.00	1.00	1.00	1.00	1.00
CMS-216	Butyl	−0.08	−0.50	−0.49	−0.27	0.09	0.21	0.82	0.99	0.91	0.94	0.95	0.89
CMS-2412	Ethyl	−0.31	−0.62	−0.53	−0.27	−0.18	0.17	0.86	0.99	0.80	0.92	0.90	0.86
CMS-2413	Methyl	−0.42	−0.68	−0.56	−0.27	−0.31	0.35	0.74	0.93	0.70	0.82	0.81	0.75

*All molecular properties are standardised against the mean and the standard deviation of a large (over 10,000 structures) dataset stored in COSMOS NG which are provided in “Step3_Properties_Info” spreadsheet of Supplementary Information.
  o Calculate the analogue quality considering the preferred selections. In this case, for overall Analogue Quality, both ToxPrints (pre-defined for interpretation) and RDKit (dynamic generation) fingerprints are used to quantify structure-based similarities combined with the Euclidean or Pearson measures of property-based similarities by taking geometric means.

**Fig. 19:**
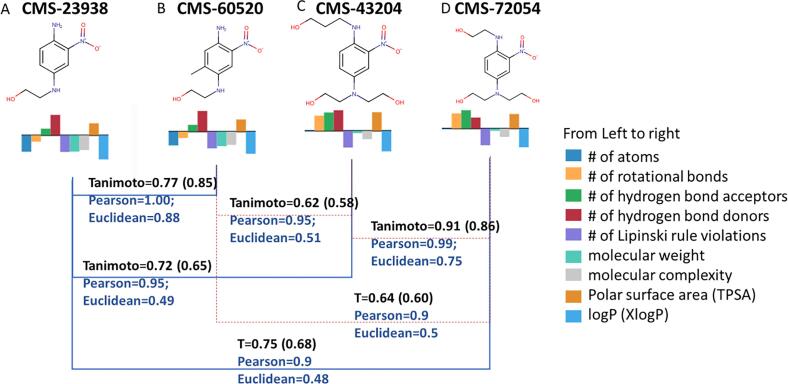
Comparison of Structure- and Property-Based Similarity. The Tanimoto coefficients in parentheses represent those from ToxPrint.

The Supplementary Information has imbedded formulae along with affiliated information needed to calculate this step of structure- and property-based similarity, leading to the analogue quality (AQ). Whilst the RDKit fingerprints and the Pearson similarity consider the three analogues quite similar to the target, ToxPrints and Euclidean Similarity seem to further differentiate the other two compounds (HC Violet No. 2; CMS-43204 & HC Blue No. 11; CMS-72054) from the target (HC Red No. 7; CMS-23938). The pair-wise comparisons are summarised in Fig. 19.

To determine potential analogues, these similarity measures are combined to result in the Analogue Quality values as given in Table 9. Note that a geometric mean was used to combine the two similarity measures (structure- and property-based) since both pieces of evidence relate to attributes of chemicals. Whilst the use of Pearson similarity with structure fingerprints give higher values than those combined with the Euclidean similarity having multiple ethoxyamine groups, only the HC Violet No. 1 (CMS-60520) has the same “aromatic primary amine ortho to nitro group” as the target. These analogue qualities are one source of evidence that will be evaluated when the decision is made for read-across based on experimental data from analogues. The Supplementary Information (Step3_Similarity and Step3_Properties_Info tabs) contains all relevant information with imbedded formulae. In this table, the option of selecting Pearson or Euclidean similarities is available.

**Table 9:** Similarity Measures and Analogue Quality.

CMS ID (Name)	Property-Based Similarity			Fingerprints-Based Similarity		Analogue Quality*	
	Euclidean distance	Euclidean similarity	Pearson similarity	ToxPrint	RDKit	AQ (Pearson)	AQ (Euclidean)
CMS-23938 (HC Red No. 7)	0	1.0	1.0	1.0	1.0	1.0	1.0
CMS-60520 (HC Violet No. 1)	0.13	0.88	0.999	0.85	0.77	0.87	0.83
CMS-43204 (HC Violet No. 2)	1.05	0.49	0.95	0.65	0.72	0.76	0.61
CMS-72054 (HC Blue No. 11)	1.08	0.48	0.90	0.68	0.75	0.77	0.63

*Analogue Quality is defined as a geometric mean of both structure- and property-based measures. The general equation (3) is reduced to $\mathrm{AQ}\;\;=\;\;\sqrt[{3}]{\mathrm{Sim}\;\left(Tox\mathrm{Pr}\mathrm{int}s\right)\;*\;Sim\left(RDKit\right)*Sim_{\mathrm{Euclidean}}\left(\mathrm{Pr}operties\right)}$.

#### Steps for chemical profiling by categories and pathways

Analogues usually are grouped within related chemical categories. In COSMOS NG, chemical categories are matched with chemotypes of liver toxicity, developmental/reproductive, and mitochondrial toxicity as well as DNA and protein binding.

• **Step 4: Calculate Chemotype Profilers Within COSMOS NG**
  o Generate “Chemotype Profiles” from the action items in the Data Table for all structures.
  o Export the Data Table again to append the number of fragment hits per profiler in the Excel Table.
  o Confirm that similar structures belong to the similar related chemical categories of the target structure in order to be considered as analogues.

Based on chemotype analysis in COSMOS NG, HC Violet No. 2 (CMS-43204) and HC Blue No. 11 (CMS-72054) were not only less similar to the target by similarity measure; but the aromatic nitro system (shaded in Table 10) also matched chemotype alerts for male reproductive toxicants [88] that were not present in the target and close analogue. The particular biological chemotype indicator argues against considering these two compounds as analogues of the target (CMS-23938). These two compounds (CMS-43204 & CMS-72054) also have lower similarity measures in Euclidean similarity and the Toxprint fingerprint-based similarity than the analogue candidate (CMS-60520). For these reasons, even if DART and Genotoxic alerts are only available in the commercial system, we can still judge by the structure- and property-based similarity measures as well as public Ashby Tennant rules in the public system. (Step 4_chemotype tab in the Supplementary Information).

**Table 10:** Chemical Chemotype Matching by Chemotypes in COSMOS NG.

Structures	Liver alerts	DNA binders	Developmental/ Reproductive alerts
CMS-23938 (HC Red No.7)	Quinones/proquinones	Aromatic nitro, amines	None
CMS-60520 (HC Violet No. 1)	Quinones and pro-quinones	Aromatic nitro, amines	None
CMS-43204 (HC Violet No. 2)	Quinones and pro-quinones	Aromatic nitro, amines	Aromatic nitro system; reproductive toxicant
CMS-72054 (HC Blue No.11)	Quinones and pro-quinones	Aromatic nitro, amines	Aromatic nitro system; male reproductive toxicant

#### Steps to find toxicity data for analogues

The next step is to line up the evidence: structure- and property-based similarities, and chemical category profiling. In this summarised table, experimental data can be attached along with the quality measures (including COSMOS MINIS score) that can lead to the Study Reliability Likelihood.

• **Step 5: Attach Appropriate Toxicity Endpoint Data And Study Reliability**
  o Find toxicity data and the MINIS Grade for each analogue candidate from the COSMOS DB.
  o Enter summarised toxicity information as well as MINIS Grade and the Opinion Score (from toxicologists if possible).
  o Calculate the Study Reliability Likelihood based on MINIS Grade and Opinion Score.

Again, the toxicity data can be either entered in the Data Table within COSMOS NG or in the exported Excel table along with the study quality measures. The “Step5_studyReliabl” tab within the file Supplementary Information represents the actions needed in this step. If no MINIS Grade is assigned in the COSMOS DB, users can estimate them by following the Appendix 1 and Table 6.

#### Steps to set up an evidence table for read-across assessment

The evidence matrix is depicted in Table 11, from which a weight-of-evidence combination can be executed in order to determine whether a similar structure can be qualified to an analogue and if so, whether the experimental data are good enough to be used for read-across.

• **Step 6: Weight-of-Evidence Table: Read-Across Reliability**
  o Prepare a “Weight-of-Evidence Table” listing all the evidence such as similarity, pathway or category profiles, if any, and toxicity data along with the study reliability. (Step6_Weight_Of_Evidence_Table in Supplementary Information).
  o Fill in Analogue Quality and Study Reliability Likelihood from the previous step. (These two quantities are highlighted in blue in Table 11.)
  o Calculate the Read-Across Reliability by getting a joint probability of the Analogue Quality and Study Reliability Likelihood.

**Table 11:** Weight-of-Evidence Table to Assess Read-Across Reliability and NOAEL Bounds.

Evidence	CMS-23938	CMS-60520	CMS-43204	CMS-72054
Structure-Based Similarity RDKit Tanimoto Coeff. ToxPrint Tanimoto Coeff.	1 1	0.77 0.85	0.72 0.65	0.75 0.68
Property-Based Similarity Euclidean similarity Pearson similarity	1 1	0.88 0.999	0.48 0.95	0.48 0.90
Chemical Category Profile Liver DNA binders Developmental/Reproductive	YES YES NO	YES YES NO	YES YES YES	YES YES YES
Analogue Quality Euclidean similarity Pearson similarity [42]		0.83 0.87	0.61 0.76	0.63 0.77
Compound Role	Target	Analogue	Similar	Similar
Toxicity Data Study Design	Assumed no data	90-day SD rat, oral-gavage/ intubation; 50–500 mg/kg-bw/day (mkd)	90-day SD rat, oral-gavage/ intubation; 50–800 mg/kg-bw/day (mkd)	90-day Wistar rat, oral-gavage/ intubation; 50–160 mg/kg-bw/day (mkd)
Study Results		Clinical signs, Clin Chem, Organ Weight dec, leading to liver pathology at higher dose NOAEL = 17 mkd (LOAEL = 50 mkd)	No adverse effect NOAEL = 50 mkd	No adverse effect NOAEL = 80 mkd
Study Source Study Quality: COSMOS MINIS Study Quality: Opinion Score Study Reliability Likelihood*	NA NA NA NA	SCCS 4 4 **0.9**	SCC 4 4 **0.9**	SCCP 4 4 **0.9**
**Read-Across Reliability** Simple Estimation** Dempster-Shafer Rules [99]		**0.75** **0.79 – 0.96**	0.55 0.52 – 0.81	0.56 0.55 – 0.83
**Read-Across Endpoint Value:** NOAEL bounds***	**14 – 33 mkd** at 95% confidence interval			

*The study reliability likelihood values were taken from Table 6.

**Property-based similarity based on Euclidean distance method was used for Analogue Quality. The estimated Read-Across Reliability values were calculated as joint probabilities between the two independent sources of evidence, i.e., Analogue Quality and Study Reliability Likelihood.

***NOAEL bounds were estimated assuming a normal distribution of NOAEL values of the nearest neighbors of the 1357 NOAEL dataset [100].

In the weight-of-evidence table, each piece of evidence is associated with a quantitative measure, from which the final Read-Across Reliability can be derived. Although in this case only one study was used per analogue, objective reliability scores of multiple studies can be used to help the selection of the most reliable data for each analogue, thus eventually decreasing the uncertainty of the read-across. For the assessment of Read-Across Reliability using the commercial system, each evidence metric can be combined by a robust approach based on Dempster Shafer Theory (DST) [99]. However, in the COSMOS NG, when combining different types of evidence, a simple joint probability will often suffice, especially for cases where there is one study and one analogue for the target. For example, in this particular case, the Read-Across Reliability of 75% can be calculated by a simple joint probability between the Analogue Quality (83% based on Euclidean distance) and Study Reliability Likelihood (90%). If either of these measure is below 70%, it is unlikely that the case is feasible or reliable for read-across. If the Study Reliability Likelihood is below 0.5, it becomes clear that this piece of data does not warrant a read-across even if used in the analysis. When the DST method was applied to obtain the Read-Across Reliability and uncertainty in a more rigorous way, the bounds were estimated at 0.79 – 0.96 with the uncertainty of 17%. This result again confirms that for the case of one analogue/one study, a simple joint probability may be a quick indicator, although it yields the most conservative estimation.

• **Step 7: Weight-of-Evidence Table: NOAEL Estimation with Data from COSMOS NG**
  o Download the NOAEL/LOAEL dataset of 1357 chemicals [100].
  o Find structural nearest neighbors of this target (CMS-23938) at 70% using ToxPrint fingerprints from the NOAEL/LOAEL dataset.
  o Calculate mean, standard deviation.

The last step in the read-across workflow is to estimate the expected toxicity measure of the target based on selected analogue(s). Although the NOAEL value of the analogue (CMS-60520), i.e., 17 mg/kg/day, may be used as a surrogate for the target, no confidence interval or insights on uncertainty can be provided since the result was derived from only one value from one structure. Since the uncertainty involved in this value is highly desired, this study uses another simple but interpretable process of estimating the NOAEL values using a NOAEL/LOAEL dataset provided within COSMOS NG. A public dataset of the new TTC dataset of 1357 chemicals enriched in antimicrobials and cosmetics can be used as a source for qualified NO(A)EL/LO(A)EL values [100]. Searching for the nearest neighbor structures (using only structure-based similarity for a rough estimate) in this dataset for the target yielded 17 similar structures around the neighborhood of the aromatic amine/nitro system for hair dyes and 11 when removing the structures hitting the reproductive toxicant profile (as discussed in Step 4, Table 10). The NOAEL bounds at 95% were roughly estimated (by assuming normal distribution) to be 14–33 mg/kg-bw/day. This rough estimation of the target structure is remarkably close to the 1-generation oral reproductive study in rats (used in margin-of-safety, MOS assessment by SCCP) based on systemic effect. Development of various methods to estimate NOAEL values within the read-across workflow were already published using the same 1357 NO(A)EL/LO(A)EL dataset [42]. This simple case illustrates the utilitarian value of the COSMOS DB and NG as part of the read-across workflow, whereas additional well-defined analysis can be added to arrive at an assessment outcome.

### COSMOS next generation as the foundation for a public knowledge hub

A knowledge hub is a network dedicated to capture, share and exchange data across multiple knowledge sources to serve diverse users. By “data”, we include interfacing framework comprised of four component types that will become the foundation: content data, models, tools, and methods.

The COSMOS NG quest for new data is ongoing. A number of additional datasets are scheduled to be imported as a part of the content update cycle. In addition to FDA CERES public content, successful results of the data exchange program enabled inclusions from the HESS database from Japanese National Institute of Technology and Evaluation (NITE) [19], OpenFoodTox [16], [17] from EFSA, ToxRefDB [18] from US EPA, Cosmetics Safety data from Korean Cosmetics Institute of Industries (KCII), and Cosmetics Europe. This collaboration formed a foundation of information sources. Also included are the datasets assembled as a part of the project such as the new TTC dataset for cosmetics and antimicrobials and the COSMOS Non-Cancer TTC dataset.

Currently COSMOS NG handles external public models and workflows by servicing from web services within the COSMOS NG framework. This framework will also communicate and support a diverse set of *in silico* tools and methods through the Knowledge Hub. One intriguing possibility is the ability to share a workflow object containing the analysis of safety/risk assessment across multiple entities. While allowing users to leverage both public and commercial systems, such a workflow could integrate information and feedback from regulatory agencies with the same from industry and academic groups. For example, the case study presented in this study could be shared across multiple institutions, enabling each to contribute to and benefit from the analyses.

## Conclusions

The COSMOS Project activities covered three main areas in the management of chemical toxicity data: an overview of toxicity data sources and studies; the design of the data model and the data entry tool for the database; and the definition of a data curation strategy. Whilst the COSMOS DB was initially a database offering the public a web-based searching/retrieval system, the new COSMOS NG contains not only the database, but also tools to calculate molecular properties and structural profilers. It also serves as a forum for sharing resources and models in support of workflow developments. The database is “compound-centred” and contains inventories from numerous regulatory programs including cosmetics inventories. For biological data, the database supports repeated dose toxicity as well as numerous other endpoints. The data have been collected, curated, quality-controlled, stored and managed in a flexible and sustainable manner to support predictive modelling tasks. An effective curation strategy for toxicity data has also been reflected in building the data entry system. Based on the review of existing approaches on good practice to assess quality entries, the reliability of the toxicity data is supported by all available data from multiple sources based on COSMOS MINIS criteria. It also provides ways to add expert opinions in the data table such that study reliability can be estimated quantitatively in the read-across workflow. COSMOS NG provides multiple fingerprinting schemes to calculate the structure-based similarity. In addition, the functionality of calculation of molecular properties enables users to include property-based similarity when searching for analogues. COSMOS NG offers several tools for compiling the data to build the evidence table. A case study of read-across analysis, taking users step-by-step through the approach using data from COSMOS NG, was demonstrated to generate a transparent read-across assessment workflow. The current design and implementation of COSMOS NG will further allow for the building of robust public safety assessment knowledge hub with qualified data in this intriguing era of big data and artificial intelligence.

## Declaration of Competing Interest

The authors declare that they have no known competing financial interests or personal relationships that could have appeared to influence the work reported in this paper.

## Figures and Tables

**Table:** 

	Data element	Description	Required?
1	STUDY INFO_TEST SUBSTANCE: NAME SOURCE	controlled vocabulary pick list of test substance names source including “unknown”	Aspect 1: OECD Guide REQUIRED
2	STUDY INFO_STUDY BACKGROUND: GUIDELINE	controlled vocabulary pick list of guideline names including non-guideline, and unknown	Aspect 1: REQUIRED
3	STUDY INFO_STUDY BACKGROUND: REFERENCE TYPE	controlled vocabulary pick list of reference type names, e.g., SCCS study report, ECHA SR DB study report, NTP study report, literature publication, etc.	Aspect 1: REQUIRED
4	STUDY DESIGN_STUDY: STUDY TYPE	controlled vocabulary pick list of subacute (≤28 days), subchronic, chronic, reproductive/ developmental, carcinogenicity (non-neoplastic) studies	Aspect 2 REQUIRED
5	STUDY DESIGN_STUDY: DURATION	number - duration days appropriate for each study types	Aspect 2: REQUIRED
6	STUDY DESIGN_TEST SYSTEM: SPECIES	controlled vocabulary pick list of the allowed species for each study type	Aspect 2: REQUIRED
7	STUDY DESIGN_ROUTE EXPOSURE AND DELIVERY METHOD	controlled vocabulary pick list of oral-dietary, oral-drinking water, oral-gavage/intubation/capsule	Aspect 2: REQUIRED
8	STUDY DESIGN: ANIMAL NUMBER	number - number of animals used at each dose and sex group	Aspect 2: NOT REQUIRED
9	STUDY DESIGN: CONTROL SUBSTANCE & DESIGN	controlled vocabulary pick list of solvent, vehicles, shams, untreated (naïve), etc. Number of animals in control.	Aspect 2: NOT REQUIRED
10	STUDY DESIGN: DOSE UNIT	controlled vocabulary list of the unit used in the treatment delivered through food, water, or gavage	Aspect 2: REQUIRED
11	STUDY DESIGN: DOSE VALUES	number - numeric value of the treatment (preferred mg/kg-bw/day conversion)	Aspect 2: REQUIRED - ON/OFF
12	RESULTS: FOOD/WATER CONSUMPTION	controlled vocabulary effects list - required for oral-dietary and oral-drinking water studies	Aspect 2: REQUIRED
13	RESULTS: BODY WEIGHT	controlled vocabulary effects list - body weight measured by the study protocol, exact numeric values are not required.	Aspect 2: REQUIRED
14	RESULTS: CLINICAL SIGNS	controlled vocabulary effects list - signs of the animals from the cage side observation, required for reproductive/developmental studies	Aspect 2: REQUIRED
15	RESULTS: PATHOLOGY	controlled vocabulary effects list - gross examination (including macroscopic pathology) and histopathology (micro), required for all doses; the organ list depends on the study types	Aspect 2: REQUIRED
16	RESULTS: ORGAN WEIGHT	controlled vocabulary effects list - organ weight measured at necropsy, the organ list depends on the study types	Aspect 2: REQUIRED

## Appendix A

Sixteen Rules Used for MINIS Grade Assignment of COSMOS DB (adapted from Yang et al., 2017)

• Urinalysis, hematology, clinical chemistry measurements are not always required in the short term studies
